# Development of effective tumor immunotherapy using a novel dendritic cell–targeting Toll-like receptor ligand

**DOI:** 10.1371/journal.pone.0188738

**Published:** 2017-11-30

**Authors:** Nadeeka H. De Silva, Takashi Akazawa, Viskam Wijewardana, Norimitsu Inoue, Maremichi Oyamada, Atsuko Ohta, Yuki Tachibana, Daluthgamage Patsy H. Wijesekera, Mitsuru Kuwamura, Yasuko Nishizawa, Kazuyuki Itoh, Takeshi Izawa, Shingo Hatoya, Tetsuya Hasegawa, Jyoji Yamate, Toshio Inaba, Kikuya Sugiura

**Affiliations:** 1 Department of Advanced Pathobiology, Graduate School of Life and Environmental Sciences, Osaka Prefecture University, Izumisano, Japan; 2 Department of Tumor Immunology, Osaka International Cancer Institute, Osaka, Japan; 3 Kakogawa Animal Hospital, Kakogawa, Japan; 4 Department of Integrated Structural Biosciences, Graduate School of Life and Environmental Sciences, Osaka Prefecture University, Izumisano, Japan; 5 Research Institute, Nozaki Tokushukai, Daitou, Japan; Ohio State University, UNITED STATES

## Abstract

Although dendritic cell (DC)-based immunotherapy shows little toxicity, improvements should be necessary to obtain satisfactory clinical outcome. Using interferon-gamma injection along with DCs, we previously obtained significant clinical responses against small or early stage malignant tumors in dogs. However, improvement was necessary to be effective to largely developed or metastatic tumors. To obtain effective methods applicable to those tumors, we herein used a DC-targeting Toll-like receptor ligand, h11c, and examined the therapeutic effects in murine subcutaneous and visceral tumor models and also in the clinical treatment of canine cancers. In murine experiments, most and significant inhibition of tumor growth and extended survival was observed in the group treated with the combination of h11c-activated DCs in combination with interferon-gamma and a cyclooxygenase2 inhibitor. Both monocytic and granulocytic myeloid-derived suppressor cells were significantly reduced by the combined treatment. Following the successful results in mice, the combined treatment was examined against canine cancers, which spontaneously generated like as those in human. The combined treatment elicited significant clinical responses against a nonepithelial malignant tumor and a malignant fibrous histiocytoma. The treatment was also successful against a bone-metastasis of squamous cell carcinoma. In the successful cases, the marked increase of tumor-responding T cells and decrease of myeloid-derived suppressor cells and regulatory T cells was observed in their peripheral blood. Although the combined treatment permitted the growth of lung cancer of renal carcinoma-metastasis, the marked elevated and long-term maintaining of the tumor-responding T cells was observed in the patient dog. Overall, the combined treatment gave rise to emphatic amelioration in DC-based cancer therapy.

## Introduction

The immune system has the potential to eliminate tumor cells by the interplay between innate and adaptive immunity. Dendritic cells (DC) are considered as the most potent antigen presenting cells to provide an essential link between innate and adaptive immunity. DC vaccination plays a major role in cancer immunotherapy, priming immune responses against cancer. Vaccination of DCs loaded with cancer antigen has given rise to some therapeutic effect in murine tumor models [1], and has been used in patients with differing types of cancer [2–4]. The treatment has almost no toxicity, but the immune responses were transient and the clinical outcome is not particularly successful. This may be partly due to degradation of DCs after injection, or inhibition of DC function by certain tumors [5,6], and various suppressor cells in the tumor environment [7]. Three improvements are required to enhance DC-based cancer immunotherapy. These are to strengthen the immune function of DCs, to improve the immune environment in cancer tissue so as to prevent degradation of DCs and facilitate the function of effector cells, and to control the generation of suppressor cells so as to maintain anti-cancer immune responses originally generated by the DCs.

Signals from Toll-like receptor (TLR) are an important link between innate and adaptive immunity. TLR 2 signals enhance the activation and maturation of DCs so as to induce antitumor cytotoxic activity [8]^.^ Post-surgery treatment with *Bacillus Calmette-Guerin* cell wall skeleton, an agonist of TLR 2, improved the prognosis of patients with lung cancer [9]. TLR 2 is expressed not only by DCs but also macrophages and some epithelial cells [10]. Also, some agonists of TLR bind nonspecifically to membranes of various cells by means of cationic charge. These properties together result in severe inflammation at the injection site. A novel synthetic lipopeptide, h11c has both a TLR2 ligand (PC2: a modified bacterial lipopeptide with two palmitate) and a DC-targeting peptide (ATPEDNGRSFS), which selectively bind to human CD11c on DCs [11]. We therefore expect h11c to give rise selectively to a potent immune response against tumor antigens presented by DCs while averting nonspecific inflammation.

We recently found that interferon-gamma (IFNγ), which is a typical activator of T helper type 1 responses, induces maturation and activation of DC, and found satisfactory clinical outcomes in the treatment of dog tumors by intratumoral injection of IFNγ along with DCs [12]. Unfortunately, this treatment is difficult to use in cases of visceral metastasis. Furthermore, IFNγ induces myeloid-derived suppressor cells (MDSC) [13]. MDSC, as well as regulatory T cell (Treg), is an undesirable inhibitor of tumor immunity, and induced by prostaglandin E2, which is produced from tumor-infiltrated macrophages and is synthesized by cyclooxygenase2 (COX-2) [14]. It therefore, follows that COX-2 inhibitor (COX2-I) have a critical role in preventing generation of MDSCs.

In this study, we show that effective therapeutic responses obtained in DC-based therapy using a combination of h11c, IFNγ and a COX2-I in mouse models of visceral tumor and in clinical treatment against canine tumors. These results propose a promising method for human cancer therapy.

## Materials and methods

### Animals and cell lines and reagents

C57BL/6 (B6), C3H/He (C3H) and BALB/c mice were purchased from Japan SLC Inc. (Hamamatsu, Japan). The mice were maintained under specific pathogen-free conditions. After starting experiments mice were monitored daily for weight loss, labored respiration and any sign of discomfort. There were no any unexpected deaths. The experimental endpoint was 60 days after injecting tumor cells. In the surface tumor models, mice were euthanized if the tumor mass grew greater than 20 mm. In the visceral tumor models, mice were euthanized if loss of appetite, suffering from pain or labored breathing was observed. The mice were humanely euthanized by anesthesia with sodium pentobarbital (200 mg/Kg, intraperitoneal injection) followed by cervical dislocation. Laboratory-bred beagle bitches, 4 to 8 years old, were housed and maintained according to NIH guidelines. All animals were kept in the animal facility of Osaka Prefecture University. The study protocols were approved by the animal experiment committee of Osaka Prefecture University. The clinical study was performed on dogs with malignant tumors, which had been admitted to the Veterinary Medical Center of Osaka Prefecture University (Izumisano, Japan) and Kakogawa Animal Hospital (Kakogawa, Japan). Written consent was obtained from all dog owners prior to the start of the study.

The B6-derived lymphoma cell line EL4, was obtained from the RIKEN BioResource Center (Tsukuba, Japan). Ovalbumin (OVA)-expressing EL4, E.G7-OVA and the BALB/c-derived colon carcinoma line CT26.WT, were obtained from the American Tissue Culture Collection (Manassas, VA, USA). The C3H-derived osteosarcoma line LM8 was established and maintained by K Itoh [15]. EL4, E.G7-OVA, and CT26.WT cells were maintained in RPMI 1640 supplemented with 10% FBS, 100 U/ml penicillin, and 100 μg/ml streptomycin (henceforth, RPMI medium). E.G7-OVA cells were cultured in the medium, supplemented with 500 μg/ml G418 (Nacarai Tesqu, Kyoto, Japan). LM8 cells were maintained as described previously [15]. Upon (intravenous) i.v. injection, LM8 cells induce tumors in the liver and lung of C3H. Similarly, CT26.WT cells develop tumors in the lung of BALB/c mice (S1 Fig).

The h11c and P2CSK4 lipopeptides and FITC-labeled versions of these lipopeptides were prepared as described previously [11]. OVA was purchased from Sigma-Aldrich Chemical (St. Louis, MO). It was confirmed as a preliminary that h11c was not toxic to dogs at less than 10 μg/kg.

### Preparation of DCs and tumor antigen

Mouse DCs were prepared from bone marrow cells, as described previously [16]. Dog DCs were prepared from peripheral blood monocytes (PBMCs) as described previously [12].

The tumor lysate was used for tumor antigens, and was prepared by the freeze- thaw method. OVA was used as the antigen of EG.7-OVA. The antigens were adjusted at 1 mg/ml in OPTI-MEM (Invitrogen).

### Assay of immune activity

Activation of mouse DCs was evaluated with expression of costimulatory molecules and cytokines by flow cytometry (FCM) or enzyme-linked immunosorbent assay (ELISA) as described previously [11].

Activity of mouse DCs to induce antigen-specific cytotoxic T lymphocytes (CTL) was evaluated in a ^51^Cr released assay as described by Koizumi et al [17]. where ^51^Cr–labeled E.G7-OVA or EL4 cells were incubated with cells from the popliteal lymph node of B6 mice after inoculation of the OVA-loaded DCs (2×10^5^) into the footpad.

To estimate the immune status of tumor tissues, immunohistochemistry (IHC) staining was preformed to detect macrophages/DCs, CTL/natural killer (NK) cells and Tregs in the tumor tissues.

Activation of dog DCs by h11c was evaluated by measuring interleukin (IL)-12 production as described previously [11]. As described by Ning et al [18], these tumor-responding T cells (TRTC) in dog PB mononuclear cells were quantified as the IFNγ-producing CD4^+^ or CD8^+^ cells by FCM after incubating with the tumor antigens (0.05–0.1 mg protein in 1 ml) at 37°C overnight.

### FCM, ELISA and IHC

For the surface staining, cells were incubated at 4°C with the following antibodies: FITC-conjugated and PE-conjugated anti-mouse CD11b mAb (clone M1/70), PE-anti-mouse CD11c mAb (clone N418), APC-conjugated anti-mouse Ly6C mAb (clone HK1.4), PE-anti-mouse Ly6G mAb (clone RB6-8C5), APC-anti-mouse OVA257-264 peptide bound to K-2Kb mAb (clone 25-D1.16), FITC-anti-mouse CD4 mAb (clone GK1.5), Biotin conjugated rat anti-mouse CD8 mAb (clone 53–6.7), FITC-anti-canine CD4 mAb (clone YKIX302.9), PerCP-eFluor®710 conjugated anti-canine CD8 mAb (clone YCATE55.9), and FITC-anti-canine major histocompatibility complex (MHC) class II molecule (MHC II) mAb (clone YKIX334.2) PerCP-eFlour 710 conjugated anti-mouse CD80 mAb (clone 16-10A1) and FITC-anti-mouse CD86 mAb (clone GL1) were purchased from eBioscience (San Diego CA). Alexa Fluor®647-conjugated anti human CD1a mAb (clone NA1/34-HLK), biotinylated anti-human CD 14 mAb (clone TÜK4) were purchased from Serotec Ltd. (Oxford, UK). For intracellular staining, a kit for fixation and permeabilization (Cytofix/CytopermTM, Becton Dickinson, San Diego, CA, USA) was used. FITC-anti-mouse IL-10 mAb (clone JES5-16E3, Invitrogen), eFluor®660-anti-rat IL-12 mAb (Clone C17.8,eBioscinece), PE-anti-mouse IFNγ mAb (clone XMG1.2, Invitrogen) and APC anti-mouse FoxP3 mAb (clone FJK-16s, eBioscience), which is a marker of Tregs [19], were used following 4 hr treatment with a Golgi-plug reagent brefaldin A (10μg/ml: Becton Dickinson). To detect dog Tregs, mouse and dog MDSC, incubation and analysis was performed as described respectively by Mizuno et al [20], Youn et al [21] or Goulart et al [22]. The assay for the affinity of h11c and P2CSK4, DCs and PBMCs were incubated as described previously [11]. FCM analyses were performed using a flow cytometer (FACS Calibur, Becton Dickinson or S3, Bio-Rad). Expression of the antigens was quantified as mean fluorescent intensity (MFI) using the associated software.

For evaluation of secreted cytokines in cultures, the culture supernatants were analyzed as described previously [11], using ELISA kits for mouse IL-12p40, mouse IL-6, mouse IL-10, mouse IFN-beta and dog IL-12p40 (R&D systems, Minneapolis, MN, USA).

IHC staining was performed on paraffin embedded section of tumor tissues as described previously [23], using rabbit polyclonal antibodies against ionized calcium-binding adapter molecule 1 (Iba1: Wako, Osaka, Japan), which is a marker of macrophage and dendritic cells, and enhanced in expression by activation [24, 25], human granzyme B (Spring Bioscience, Pleasanton, CA, USA), which is a marker of CTLs and NK cells [26], and mAb against mouse FoxP3 (clone FJK-16s, eBioscience). Cross reactivity of the polyclonal antibodies to the corresponding mouse antigens was confirmed by the manufacturers. The numbers of positive cells were counted in five high-power (×400) fields selected randomly, and the positive cells in a total of 1000 cells were evaluated.

### DC-based immunotherapy

In the mouse model, six treatments were prepared for examination of the therapeutic effects: (*i*) [DC], mouse DCs (2×10^6^) were suspended in 0.2 ml tumor antigen solution and incubated at 37°C for 2 hr. After incubation, the DCs were washed and suspended with 0.2 mL PBS (-) prior to inoculation. The DC inoculation was performed four times at 7 day-intervals. (*ii*) [DC]+IFN, in which, in addition to the [DC] treatment, 2 μg/kg recombinant murine IFNγ (Peprotech, Rocky Hill NJ) was subcutaneously (s.c.) injected on the day of the DC injection (day 0), and on days 2 and 5. (*iii*) [DC]+IFN+COX2-I, in addition to the [DC]+IFN treatment, a COX2-I, celecoxib (1.67 mg/kg, BioVision, Milpitas, CA) was injected on days 0, 2 and 5. (*iv*) [h11c-DC]+IFN. DCs were incubated in the tumor antigen solution along with h11c (0.2 nmol). IFNγ was injected as in the [DC]+IFN treatment. (v) [h11c-DC]+IFN+COX2-I; (v*i*) IFN+COX2-I. The treatments were performed for the following combinations of tumor and mouse strain: E.G7-OVA–B6, LM8–C3H and CT26.WT–BALB/c. As described previously [11], E.G7-OVA cells (2×10^6^) were s.c. injected on the back of B6 mice. Treatments began 10–12 days after the injection when the diameter of the tumors was 5–6 mm. DCs were injected directly into the tumors. The treatments were performed four times at 7-day intervals. The size of the tumors was measured 3 times per week, and the volume was calculated as described previously [27]. In the experiment using LM8 or CT26.WT, tumor cells (2×10^5^) were i.v. injected and DCs were s.c. inoculated. Treatments started on the same day as the injection of tumor cells, and were performed four times at 7-day intervals. The therapeutic effect was evaluated according to the survival of the mice after the injection to the tumor. The endpoint was 60 days after the treatments.

In the clinical treatments in dogs, DCs were incubated with h11c (1.2–2.4 μg/kg) in 0.5 ml solution of tumor antigens (0.5–1.0 mg/ml) at 37°C for 2 hr. After incubation the DCs were injected directly in to surface tumors or s.c. for visceral tumors, along with recombinant canine IFNγ (10^4^ unit/kg, Interdog®, Toray, Tokyo, Japan). The dose of recombinant canine IFNγ was that recommended by the manufacturer. The injection was repeated at weekly intervals. In addition, the dogs were orally administrated daily with a COX2-I, firocoxib (Previcox®, Nippon Zenyaku Kogyo, Fukushima, Japan) at the manufacture’s recommended dose. The therapeutic effect was evaluated by measuring the tumor size, and by FCM analysis for the TRTCs, Tregs (CD4^+^ FoxP3^+^) and MDSCs (CD11b^+^ CD14^-^MHC II^-^).

### Statistical analysis

Survival of different groups was analyzed by the Kaplan-Meier method and compared using the log rank test. In the other experiments, groups were compared using Student’s *t* (two parameters) or Tukey-Kramer multiple comparison test (more than three parameters).

## Results

### h11c significantly enhanced DC functions for tumor immunity

**Fig 1:**
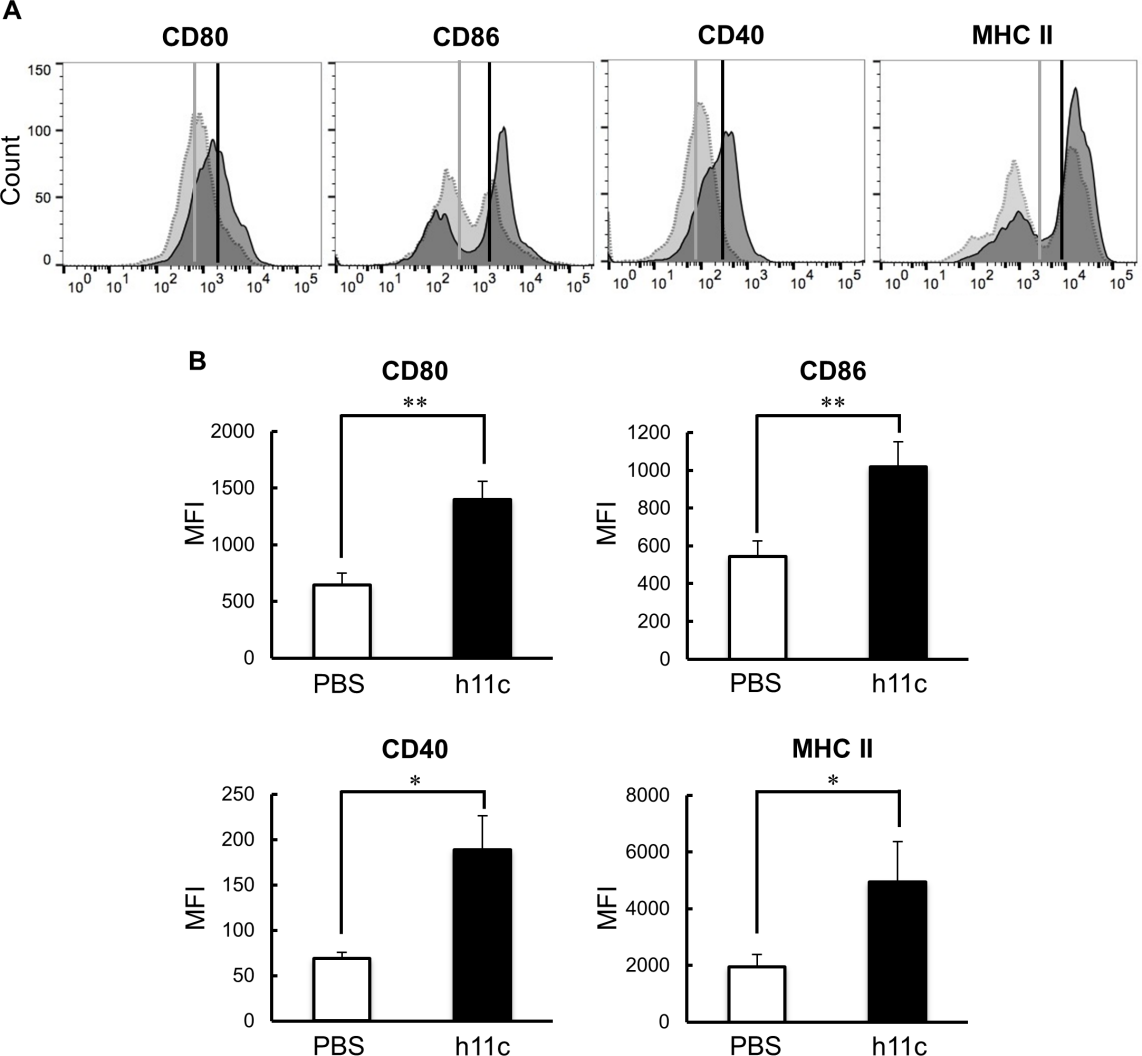
Effect of h11c on the surface molecules of DCs. DCs induced from BMCs of B6 mice were incubated with or without h11c. After incubation, the expression intensity of indicated costimulatory molecules and an antigen-presenting molecule was evaluated by FCM. (A) Representative FCM data are shown. The darker gray peaks indicate expression of surface molecules on DCs incubated with h11c. The lighter gray peaks indicate those on DCs incubated with PBS as a control. Vertical lines indicate MFI of each peak. (B) Expression intensity of surface molecules. The expression intensity of surface molecules was evaluated with MFI. Black bars indicate the expression intensity of the indicated molecules on the h11c-treated DCs. White bars indicate those of the PBS-treated DCs. Experiments were performed using five mice in each group. Results are expressed as mean ± SEM. * *p <0.01, *p <0.05.

**Fig 2:**
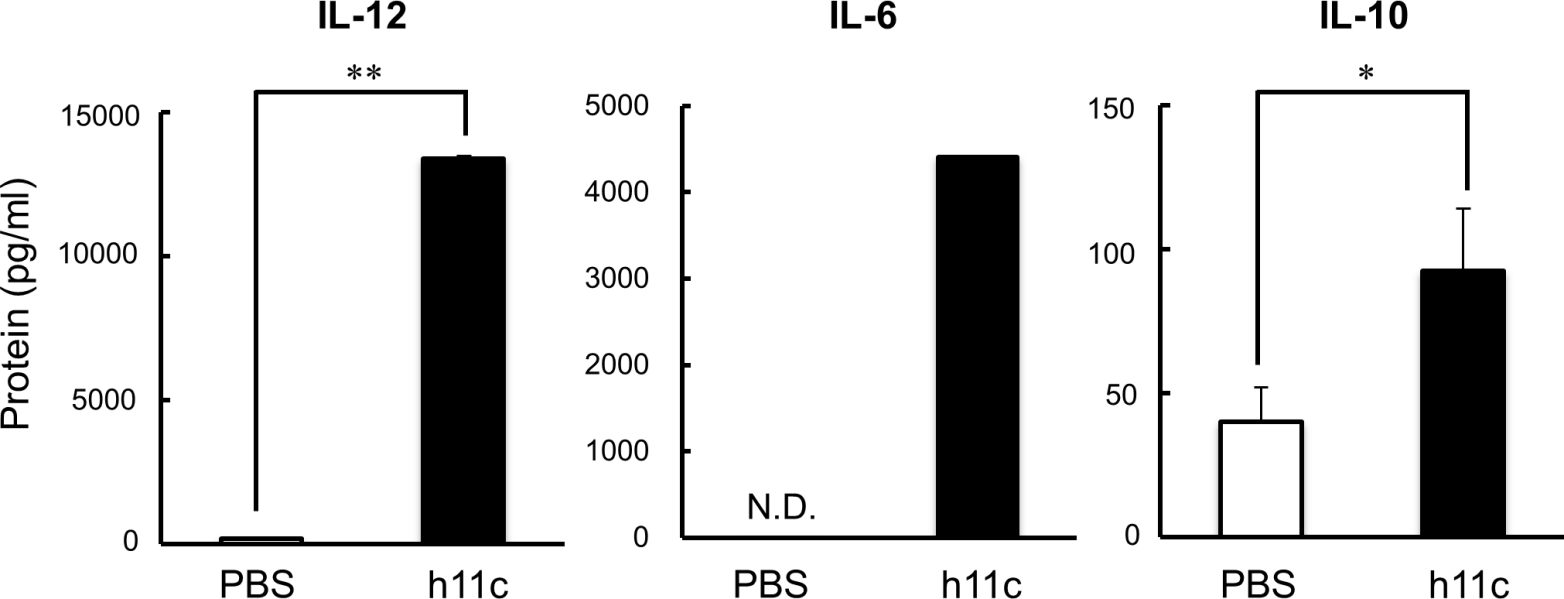
Effect of h11c on the cytokine production of DCs. DCs induced from BMCs of B6 mice were incubated with or without h11c. After incubation, expression of the indicated cytokines was evaluated by ELISA. Black bars indicate the expression intensity of the indicated molecules on the h11c-treated DCs. White bars indicate those of the PBS-treated DCs. Experiments were performed using five mice in each group. Results are expressed as mean ± SEM. * *p <0.01, *p <0.05, N.D.: not determined.

**Fig 3:**
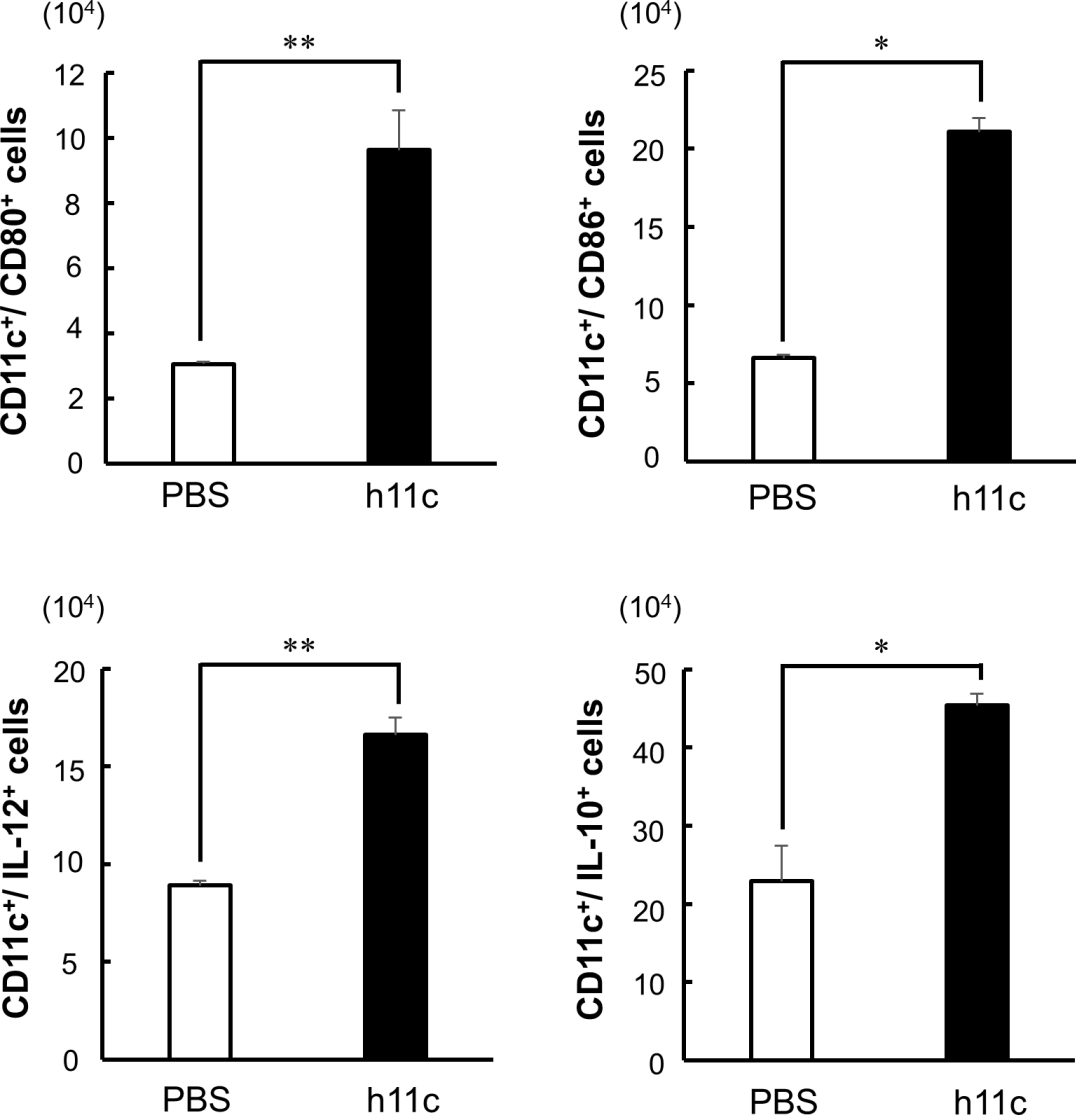
Effect of h11c on DCs of normal mice. Ten nanomol of h11c was s.c. injected into the back of normal BALB/c mice three times with a 7 day interval. Cells were collected from the inguinal and axillary LNs of the injected mice on the next day of the third treatment. The number of DCs (CD11c+ cells) expressing indicated costimulatory molecules and cytokines were evaluated. PBS was injected as a control of h11c. Experiments were performed using 3 mice in each group. Results are expressed as mean ± SEM. * *p <0.01, *p <0.05.

As shown in Fig 1, by the incubation with h11c (100 nM) for 24 hr, DCs significantly enhanced the expression of costimulatory molecules (CD40, CD80 and CD86) and a molecule for presentation of antigens (MHC II), which are required for activation of immune responses of T cells. In addition to these molecules, the incubated DCs significantly enhanced expression of IL-12 and IL-6 from DCs (Fig 2), which activate cellular and humoral immunity respectively. However, the DCs also enhanced the expression of IL-10, which suppresses immune responses. DCs expressed little amount of IFN-beta did not show any increase with h11c (data not shown). As same as the *in vitro* results, DCs collected from lymph node (LN) of mice injected with h11c significantly enhanced the expression of the costimulatory molecules and cytokines (Fig 3, S2 Fig and S3 Fig). However, the treatment with h11c did not effected on the expression of those molecules and cytokines of DCs in the spleens (data not shown).

**Fig 4:**
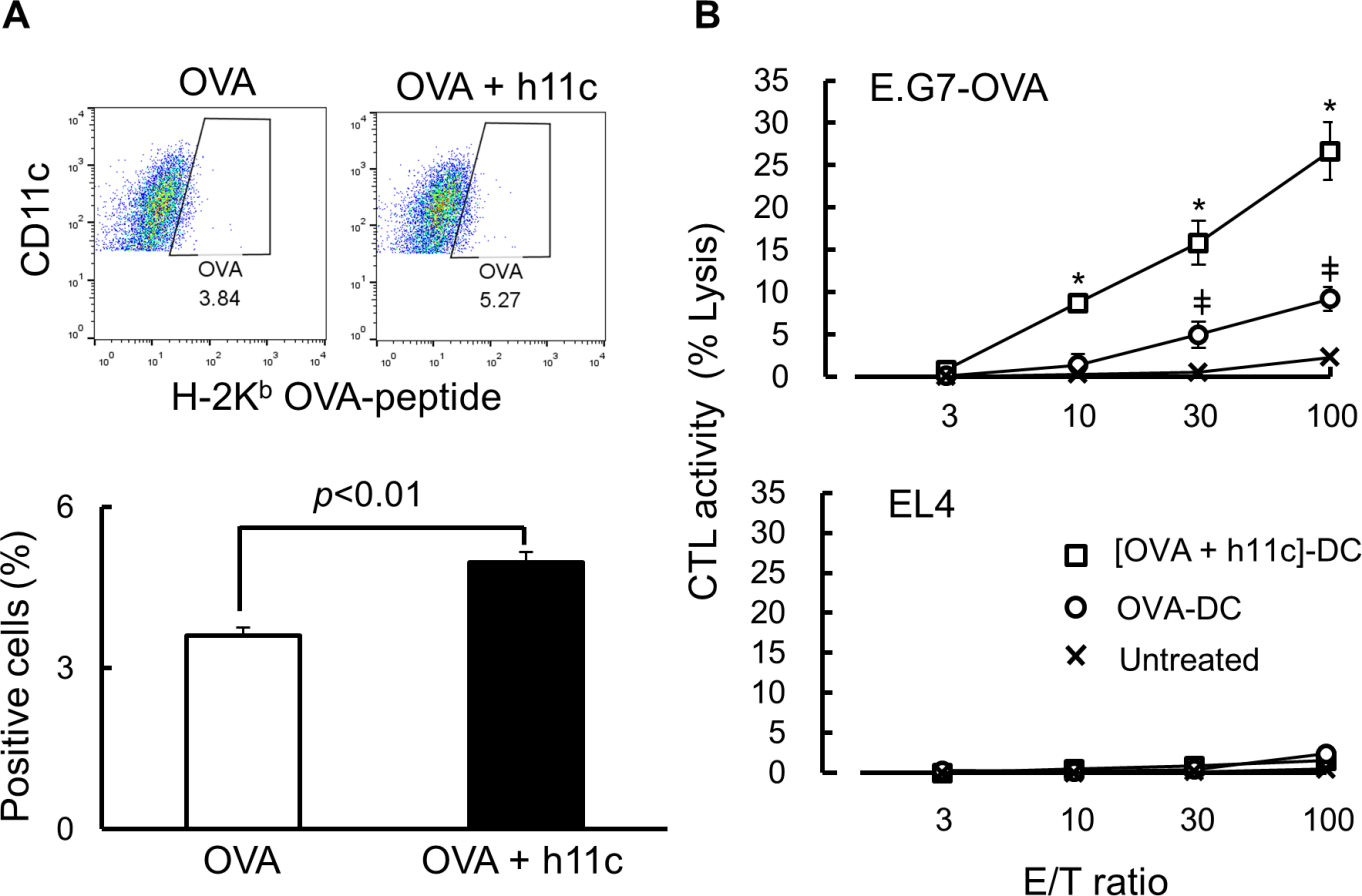
Effect of h11c on the functions of DCs derived from mouse BMCs. (A) The effect on antigen presentation. DCs from BMCs of B6 mouse were incubated with OVA in the presence or absence of h11c. After the incubation the OVA peptide presented on DCs was detected using a map against the OVA peptide expressed on the H-2K^b^ molecule. (B) Effect on the CTL activation of DC. DCs from B6 BMCs were incubated as described in (A). The incubated DCs were inoculated in to the footpad of B6 mice. After the two inoculations, cells collected from popliteal LN were incubated with ^51^Cr-labeled E.G7 or EL4 cells. The CTL activity was calculated as described in the Materials and Methods section. Experiments were performed using five mice in each group. * *p* <0.01, ^ǂ^ *p* <0.05 vs untreated group.

As shown in Fig 4A, h11c significantly enhanced the presentation of OVA peptide on MHC class I molecules of DCs incubated with OVA. Moreover, when DCs treated with OVA and h11c (OVA+h11c) were immunized, significantly higher OVA-specific CTL activity was induced, relative to DCs treated with OVA alone (Fig 4B).

### The combination of h11c-treated DC and COX-2 inhibitor significantly inhibited tumor growth and enhanced immune activity against tumor in the mouse model

**Fig 5:**
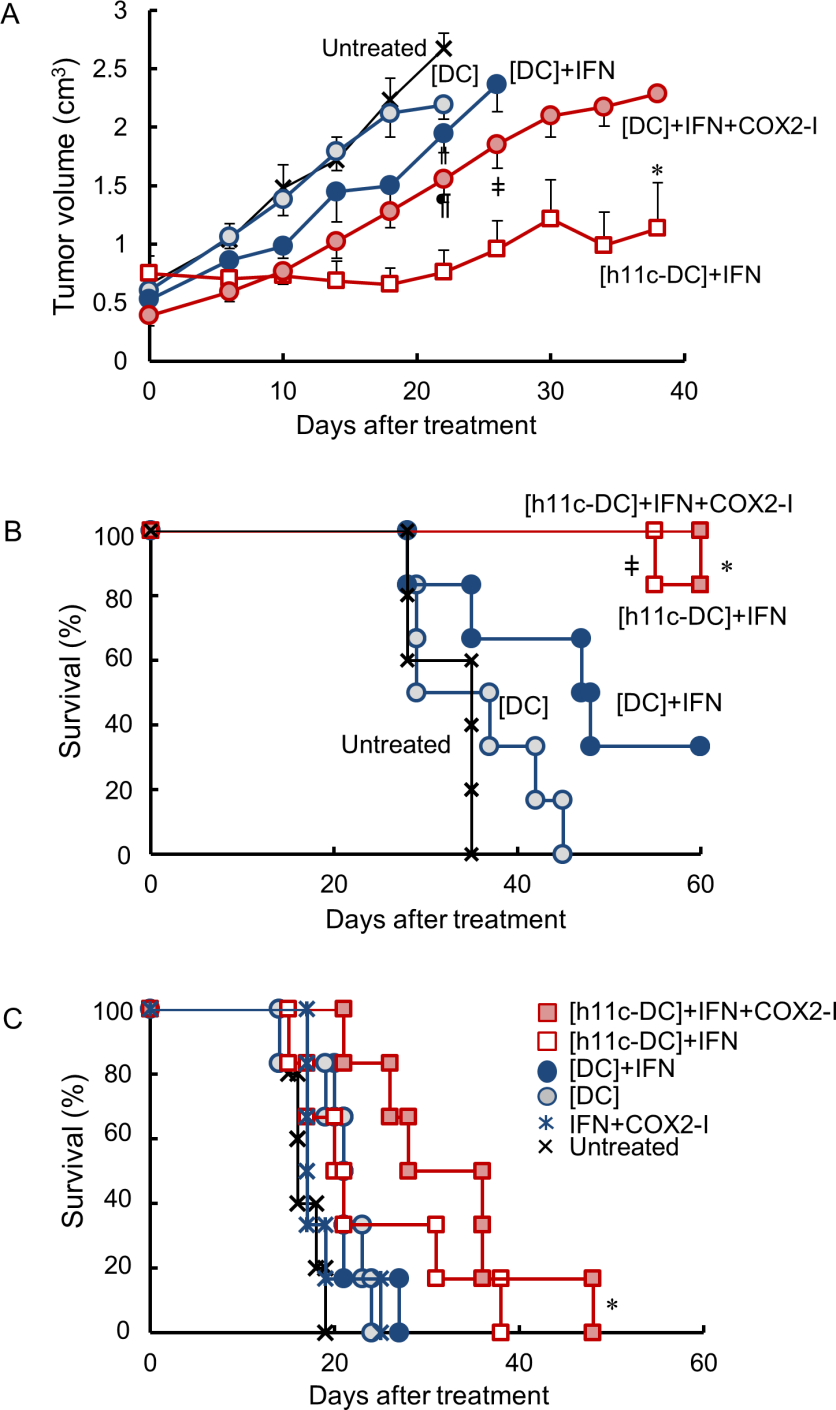
Enhancement of the antitumor effect by the treatment with h11c-treated DCs and COX-2 inhibitor. (A) The treatments indicated were performed against E.G7 tumors growing on the backs of B6 mice. The volume of tumors after the treatment is shown. Results are expressed as mean ± SEM. The experiments were performed independently three times using a total of 6 mice in each group. * *p* < 0.05 vs the [DC]+IFN+COX2-I, ǂ *p* <0.05 vs the [DC] and the untreated groups. ^¶^ *P* < 0.05 vs other groups, ^ǁ^ *P* < 0.05 vs the [DC] and the untreated group. (B) The treatments indicated were performed against LM8 cells growing in the liver and lung of C3H mice. Survival rates after the start of treatment are shown. The experiments were performed independently three times using a total of 6 mice in each group. * and ^ǂ^ *p* < 0.01 vs the [DC] and the untreated group. (C) The treatments indicated were performed against CT26WT cells growing in the lung of BALB/c mice. Survival rates after the start of treatment are shown. The experiments were performed independently three times using a total of 6 mice in each group. * *p* < 0.05 vs the other groups.

Three combinations of the tumor transplantation models in mouse were chosen in order to investigate the therapeutic effect. To estimate the local inhibition of tumor growth, the OVA antigen-presenting DCs were injected into tumors of E.G7-OVA growing on the backs of syngeneic B6 mice. As shown in Fig 5A, tumor growth was significantly inhibited by the combination of h11c-treated DC and IFNγ treatments ([h11c-DC]+IFN), relative to all other treatments. Treatment with the combination of untreated DCs, IFNγ and celecoxib ([DC]+IFN+COX2-I) tended to exert an inhibitory effect. The [DC]+IFN+COX2-I group differed significantly in tumor volume at one point (day 22), relative to the group treated with [DC] and the untreated group. No significant inhibitory effect was found in the [DC]+IFN and [DC] groups. To investigate the distant or systemic effect, the i.v. treatments of DC were administered against the visceral tumors. As shown in Fig 5B, the growth of LM8 in the liver of C3H mice, the [h11c-DC]+IFN and [h11c-DC]+IFN+ COX2-I treatments was significantly inhibited. In this group five of six mice were survived at the endpoint. Moreover, three of six mice in the [h11c-DC]+IFN+COX2-I group were tumor-free at the endpoint, together with one of the six mice in the [h11c-DC]+IFN group. The survival of the [DC]+IFN group appeared better survival (*p* = 0.085), although not statistically different, relative to the [DC] treatment and the non-treated group. In the model of CT26.WT growth in BALB/c lung, no mice treated survived by the endpoint (Fig 5C). There was a significantly better survival in the [h11c-DC]+IFN+COX2-I group than the other group, however.

**Fig 6:**
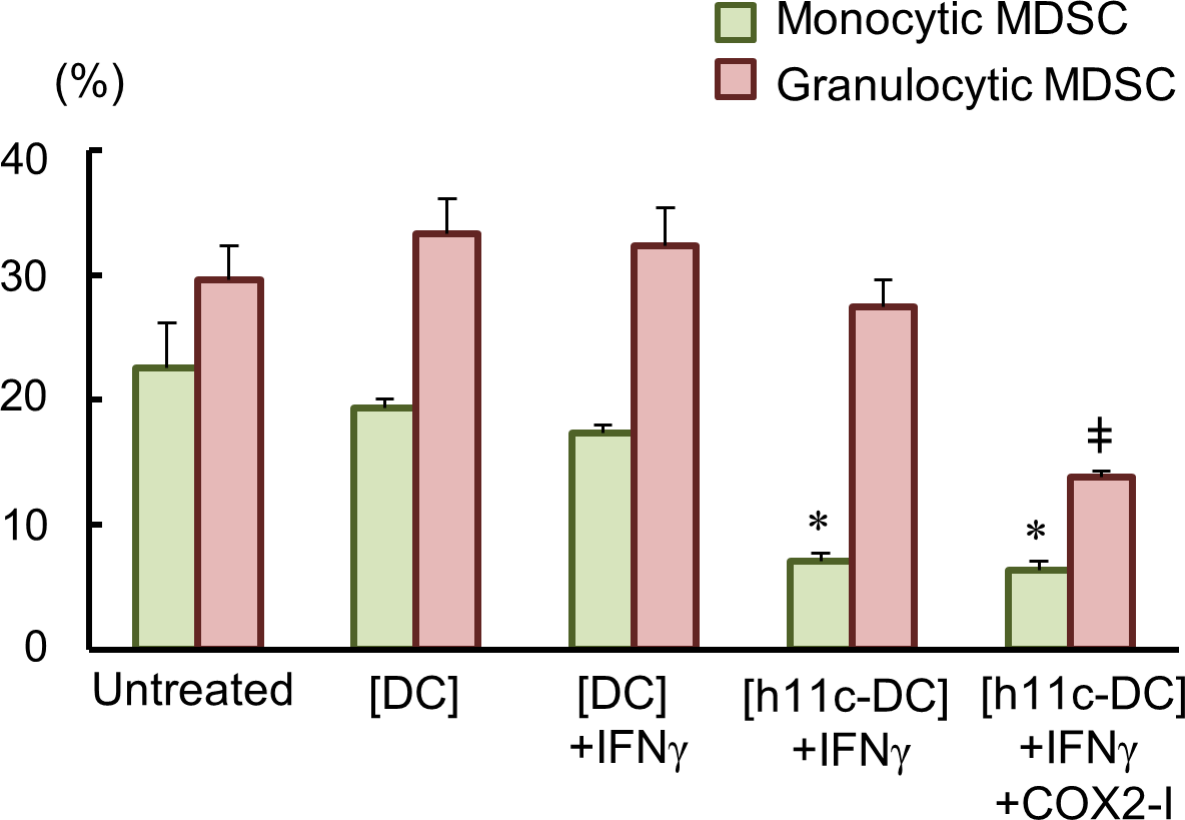
Reduction of MDSCs by the treatment with h11c-treated DCs and COX-2 inhibitor. Percentage of monocytic and granulocytic MDSCs in PB of BALB/c mice after two administrations of the treatments indicated. Results are expressed as mean ± SEM. The experiments were performed independently twice, using a total of 4 mice in each group. * and ^ǂ^ *p* <0.05 vs the other groups.

To examine that the treatment with COX2-I indeed reduces MDSCs, monocytic MDSCs (CD11b^+^, Ly6C^+^, LY6G^-^) and granulocytic MDSCs (CD11b^+^, Ly6C^+^, LY6G^+^) were evaluated two days after the second treatment (day 16) in the CT26.WT-BALB/c experiment. As shown in Fig 6, both subsets of MDSCs decreased significantly in the [h11c-DC]+IFN+COX2-I group. It is noteworthy that monocytic MDSCs unexpectedly reduced by the [h11c-DC]+IFN.

**Fig 7:**
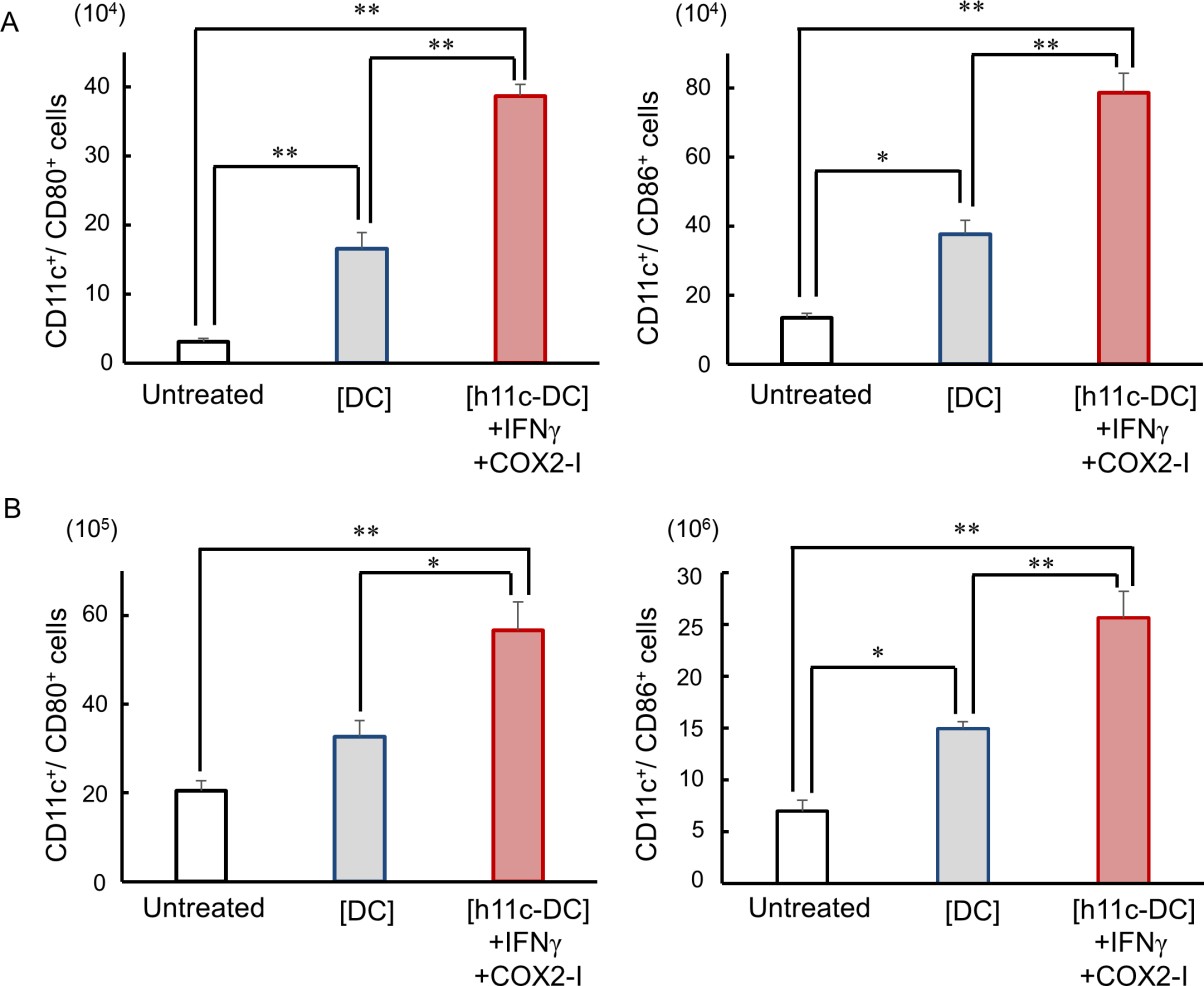
Effect of the treatments on the expression of costimulatory molecules on DCs. The treatments indicated were performed against CT26WT cells growing in the lung of BALB/c mice. Treatments were performed 3 times with a 7 day interval. Cells were collected from the inguinal and axillary LNs and spleens on the next day of the third treatment. The numbers of DCs (CD11c^+^ cells) in the LNs (A) and spleens (B) expressing indicated costimulatory molecules are shown. The FCS profiles obtained were similar to S2 Fig. Experiments were performed using 3 mice in each group. Results are expressed as mean ± SEM. * *p <0.01, *p <0.05.

**Fig 8:**
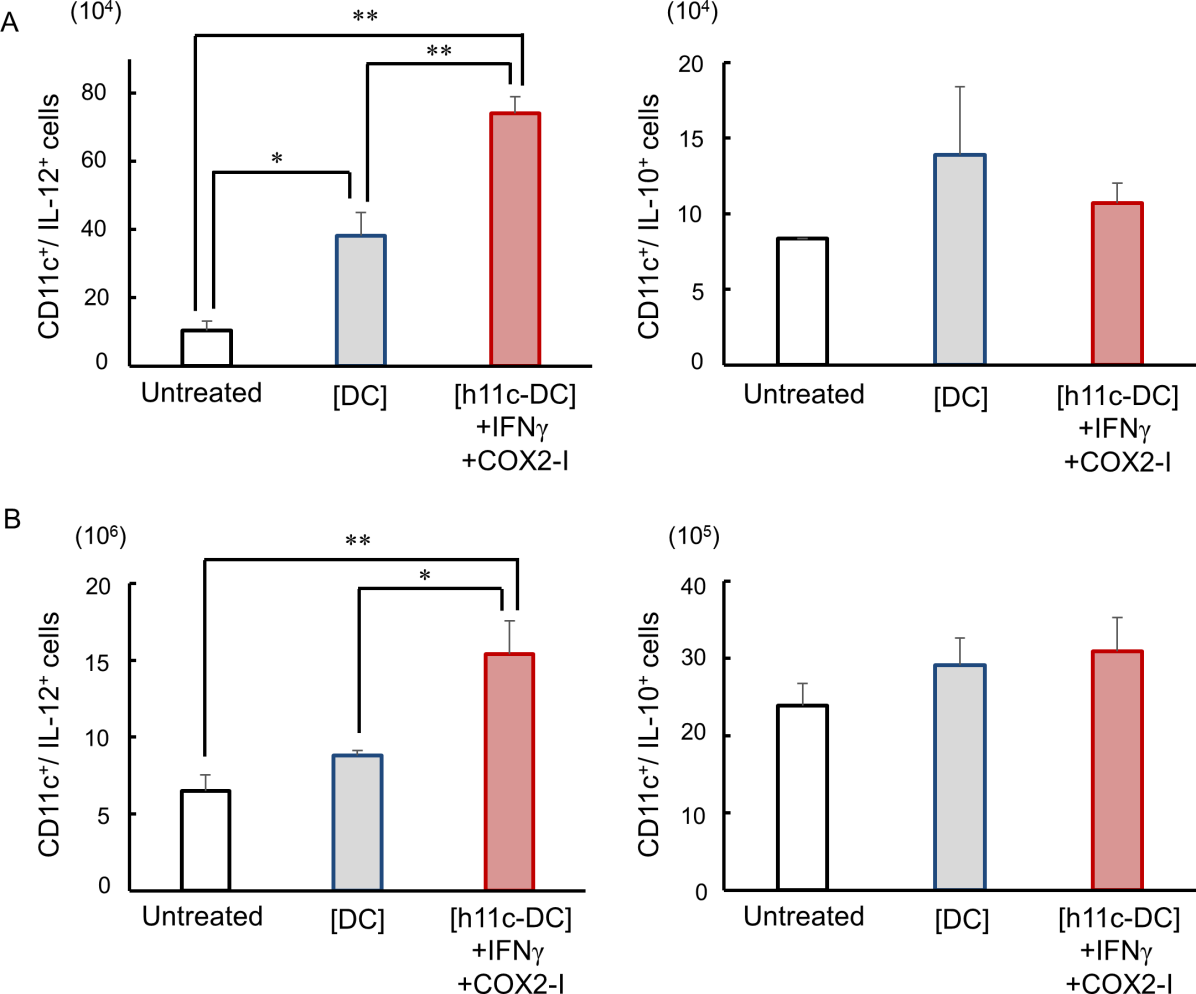
Effect of the treatments on the expression of cytokines on DCs. The treatments indicated were performed against CT26WT cells growing in the lung of BALB/c mice. Treatments were performed 3 times with a 7 day interval. Cells were collected from the inguinal and axillary LNs and spleens on the next day of the third treatment. The numbers of DCs (CD11c^+^ cells) in the LNs (A) and spleens (B) expressing indicated cytokines are shown. The FCS profiles obtained were similar to S3 Fig. Experiments were performed using 3 mice in each group. Results are expressed as mean ± SEM. * *p <0.01, *p <0.05.

Since administration of h11c into normal mice enhanced immune activation of DC, concerning expression of costimulatory molecules and cytokine, we examined the DC activity, and compared between the successful and unsuccessful regimens. As shown in Figs 7 and 8, both LN and spleen DCs in the mice treated with the [h11c-DC]+IFN+COX2-I, successful regimen showed significantly higher expression of costimulatory molecules and IL-12, which enhance antitumor immunity, compared with the DCs in the mice treated with the [DC], unsuccessful regimen and the untreated mice. However, there is no significant difference in expression of immunosuppressive IL-10 among the DCs with these treatment.

**Fig 9:**
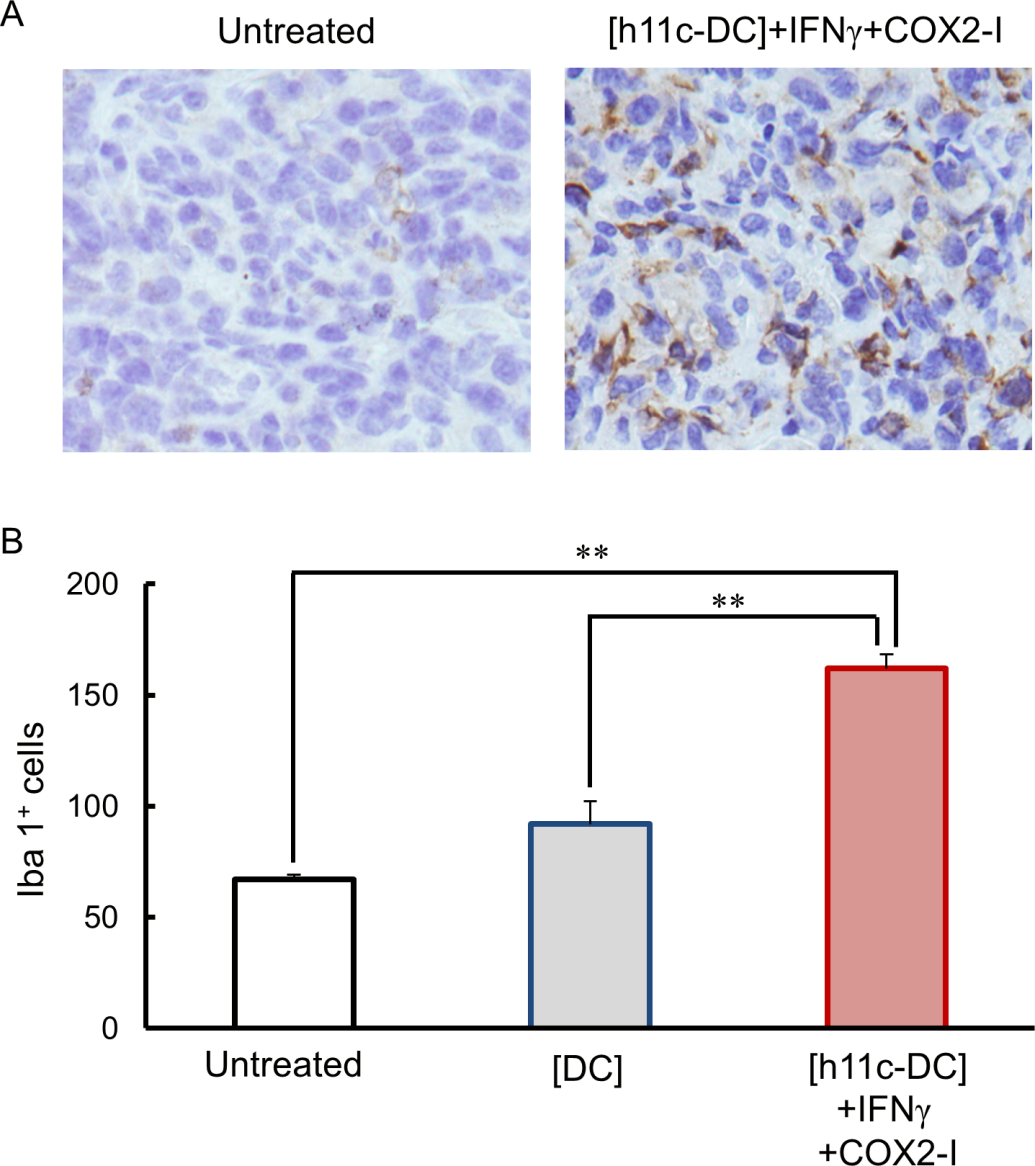
Effect of the treatments on the infiltration of macrophage/DC in the tumor tissue. The treatments indicated were performed against CT26WT cells growing in the lung of BALB/c mice. Treatments were performed 3 times with a 7 day interval. Lungs were collected on the next day of third treatment. The infiltration of the Iba 1^+^ macrophages/DCs in the tumor tissue was evaluated by IHC. (A) Representative photo data of the lung tumor of the mice with the indicated treatments are shown. (B) The numbers of the Iba 1^+^ cells per 1000 cells in the lung tumor of the mice with the indicated treatments are shown. Experiments were performed using 3 mice in each group. Results are expressed as mean ± SEM. * *p <0.01, *p <0.05.

**Fig 10:**
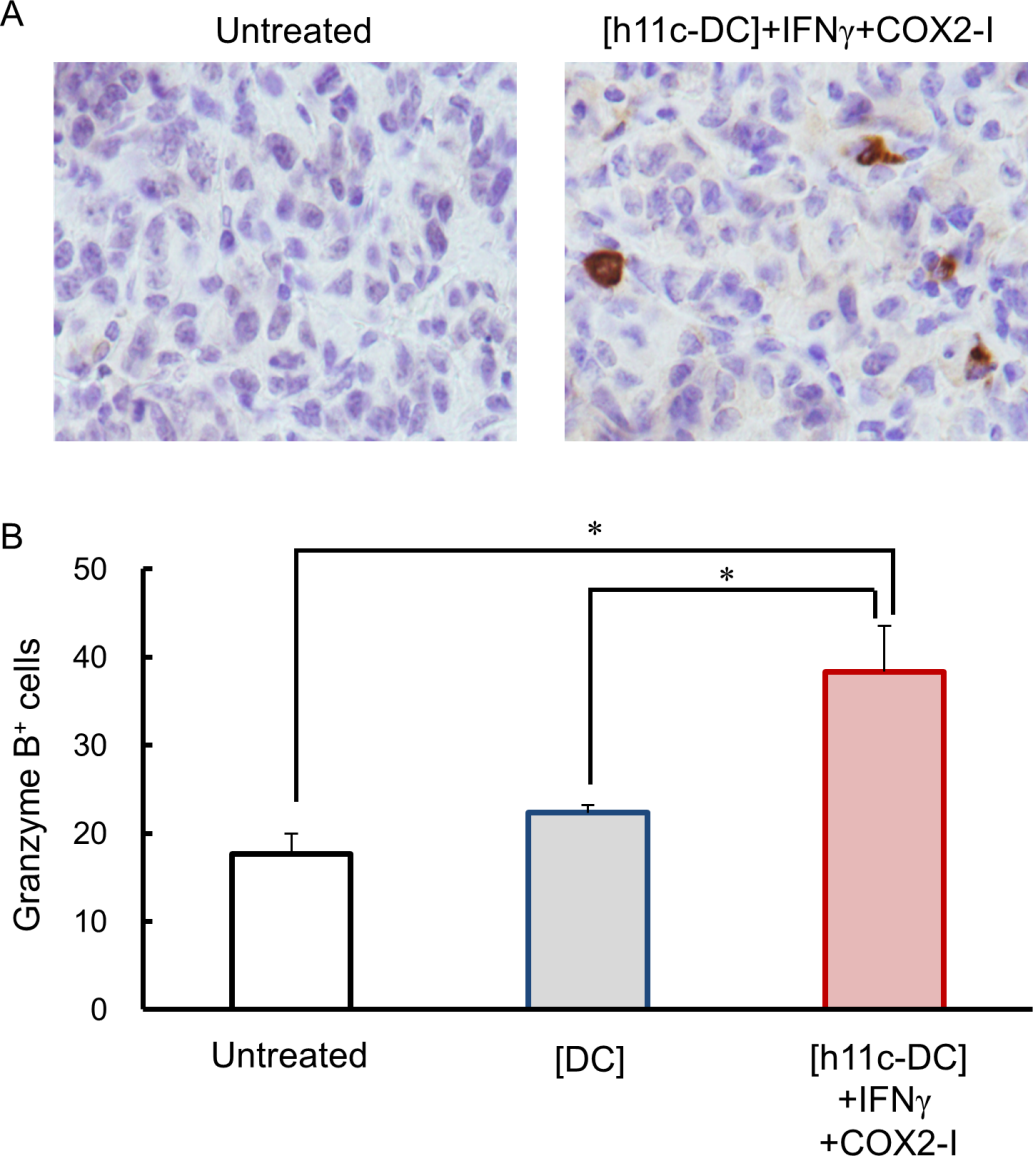
Effect of the treatments on the infiltration of CTLs/NK cells in the tumor tissue. The treatments indicated were performed against CT26WT cells growing in the lung of BALB/c mice. Treatments were performed 3 times with a 7 day interval. Lungs were collected on the next day of third treatment. The infiltration of the granzyme B^+^ CTLs/NK cells in the tumor tissue was evaluated by IHC. (A) Representative photo data of the lung tumor of the mice with the indicated treatments are shown. (B) The numbers of the granzyme B^+^ cells per 1000 cells in the lung tumor of the mice with the indicated treatments are shown. Experiments were performed using 3 mice in each group. Results are expressed as mean ± SEM. *p <0.05.

**Fig 11:**
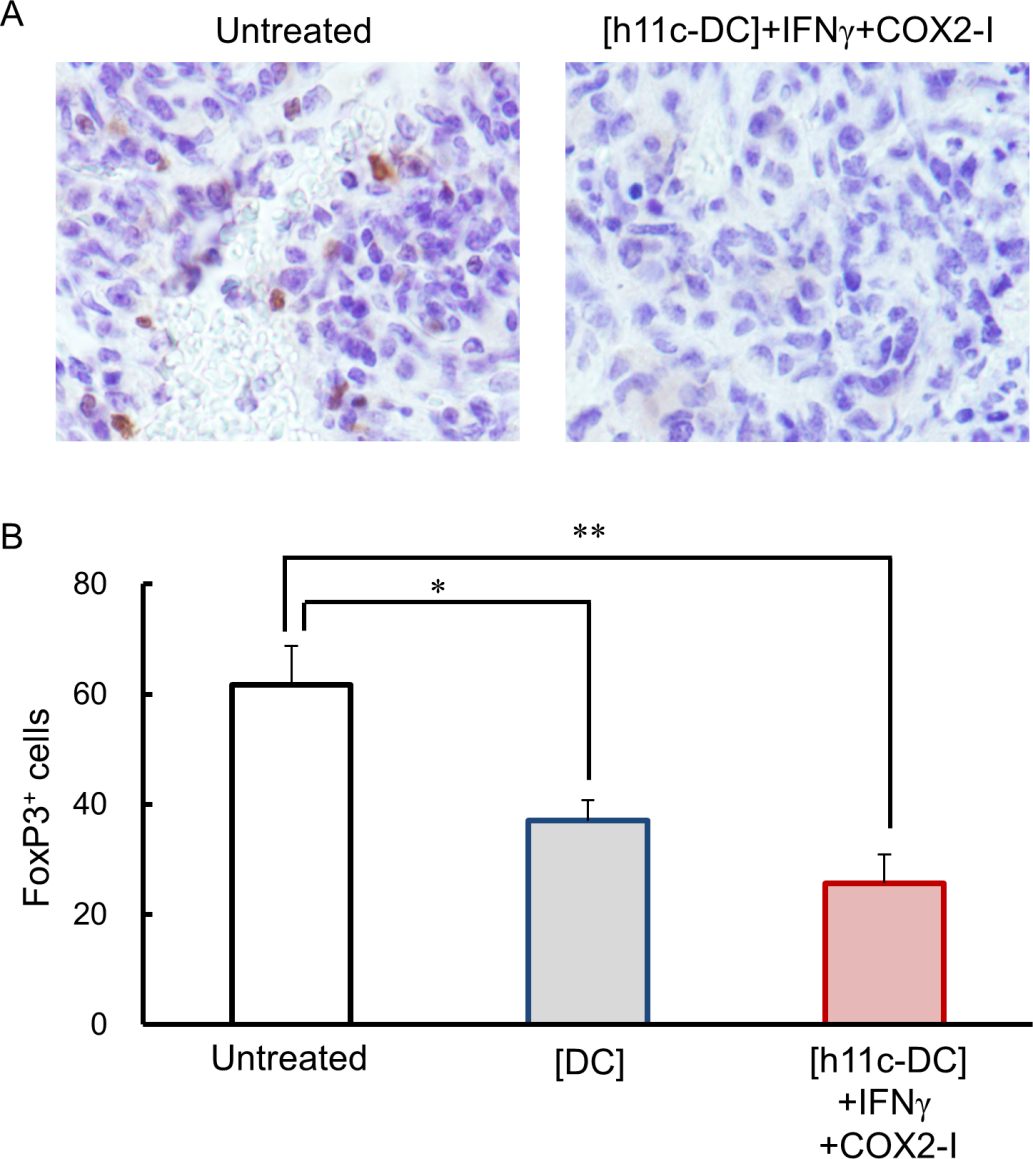
Effect of the treatments on the infiltration of Tregs in the tumor tissue. The treatments indicated were performed against CT26WT cells growing in the lung of BALB/c mice. Treatments were performed 3 times with a 7 day interval. Lungs were collected on the next day of third treatment. The infiltration of the FoxP3^+^ Tregs in the tumor tissue was evaluated by IHC. (A) Representative photo data of the lung tumor of the mice with the indicated treatments are shown. (B) The numbers of the FoxP3^+^ cells per 1000 cells in the lung tumor of the mice with the indicated treatments are shown. Experiments were performed using 3 mice in each group. Results are expressed as mean ± SEM. * *p <0.01, *p <0.05.

Finally, we estimate immune status in tumor tissues of the treated mice by evaluating the infiltrating cells. As shown in Fig 9. Iba 1^+^ macrophages/DCs were significantly more in the tissue of tumors with the [h11c-DC]+IFN+COX2-I, compared with tumors with the [DC] and untreated tumors. Similarly, significantly more number of granzyme B^+^ CTLs/NK cells were found in the tumors with the [h11c-DC]+IFN+COX2-I than those with the other treatments (Fig 10). In contrast, FoxP3^+^ Tregs were significantly lesser in the tumors with the [h11c-DC]+IFN+COX2-I and the [DC], compared with untreated tumors (Fig 11).

### h11c had an affinity for dog DCs and activated them

**Fig 12:**
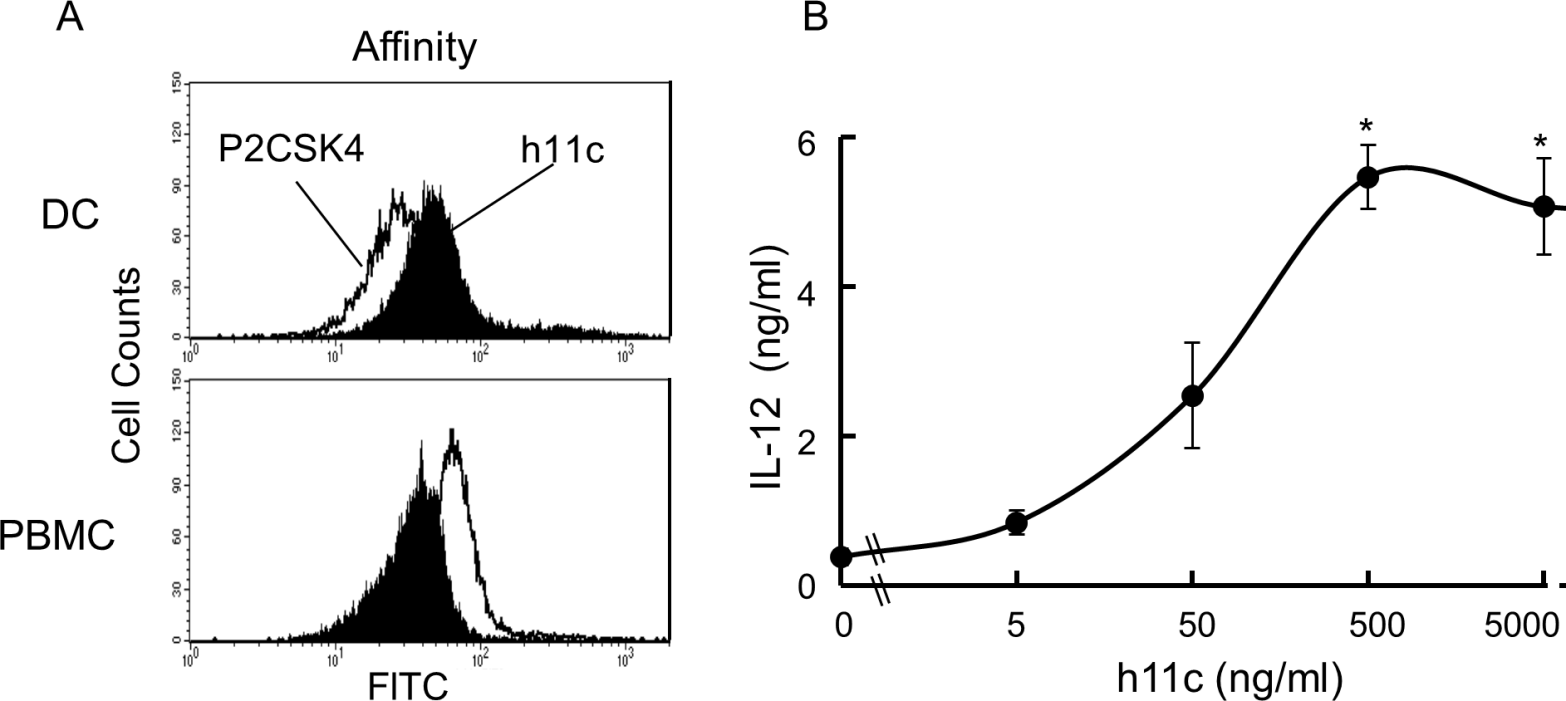
Effect of h11c on the functions of DCs. (A) The affinity of h11c and P2CSK4 to dog DC and PBMCs. Representative data from two experiments with similar results are shown. (B) Production of IL-12 from DC at the indicated concentrations of h11c. Results are expressed as mean ± SEM. The experiments were performed independently four times. * *p* <0.05 vs the other concentrations.

Since the combined treatment ([h11c-DC]+IFN+COX2-I) elicited successful results in murine tumor-models, we, as the next step, examined the enhancing effects in the clinical treatment for tumor patients of dog. The h11c was originally designed to bind to human CD11c. In a previous study it was demonstrated that h11c has high affinity for mouse bone marrow derived DCs [16]. We therefore first examined whether h11c has an affinity for dog DCs, and activates them. As shown in Fig 12A, the affinity of h11c for the dog DCs was greater than that of PC2SK4, which has the same TLR2 motif, but is a defect of the CD11c targeting molecule. In contrast, the affinity of h11c for PBMCs was less than that of PC2SK4. Moreover, as shown in Fig 12B, h11c significantly activated dog DCs in concentrations greater than 500 ng/ml.

### h11c-treatment, IFNγ and COX-2 inhibitor elicited significant clinical responses against some kinds of tumor in the dogs

**Fig 13:**
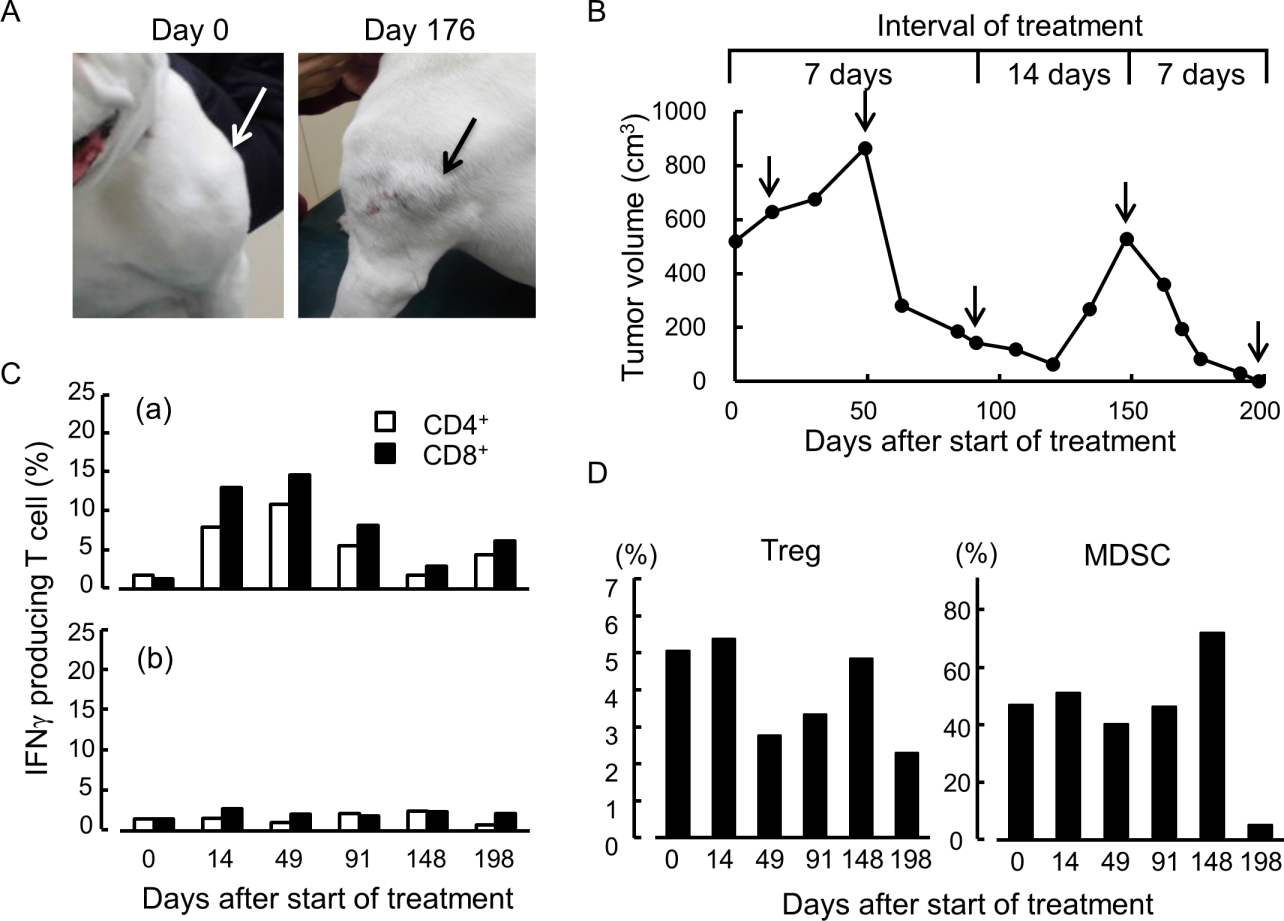
Therapeutic and analytic results for case no. 1. (A) Tumor appearance before and after the 20th treatment (day 176). (B) Volume of the tumor after the start of treatment. The points indicated with arrows are when the analyses of the PB cells were performed. (C) Responses of T cells against tumor antigens. T cells were collected from PB on the days indicated by arrows in Fig 13B. The responses against own tumor lysate are shown in (a), and those against the lysate of an unrelated tumor are shown in (b). The protein concentration of the lysate in culture was 50 μg/ml. (D) Percentage of Treg and MDSC in the PB at the points indicated.

We finally examined the therapeutic effect of the combined treatment ([h11c-DC] +IFN+COX2-I) on tumors spontaneously occurring in dogs. The characteristics of the patient dogs, their tumors and clinical outcomes are summarized in Table 1. As shown in Fig 13A, case no. 1 had a soft-ball-size tumor in the left shoulder. From histological analysis this was diagnosed as a nonepithelial malignant tumor (S4 Fig). Because this dog had a heart disorder, the owner wanted a nonoperational, DC treatment. As shown in Fig 13B, the volume of the tumor had increased by 49 days after the start of the combined treatment, but began to decrease thereafter, and by day 91 was one third of the start size. The treatment interval was then changed to one in two weeks. The tumor volume continued to decrease by day 120, but then began to increase again. As a result, from day 148, the treatment interval reverted to weekly. The tumor again began to reduce, and had almost disappeared by day 198. However, the patient dog died on day 230, due to an unrelated heart disorder. FCM analysis was performed using PB collected on days 0, 14, 49, 91, 148 and 198. As shown in Fig 13C, TRTC responses against own-tumor antigens were evaluated by IFNγ production. TRTC were first increased as shown by day 49, then decreased as shown by day 91, then finally increased by remission. The response against unrelated tumor antigens was low and did not change throughout the treatment period. As shown in Fig 13D, a reduced level of Tregs was observed on day 49, but had increased by day 148, and was lowest at the remission. The level of MDSCs changed in a similar manner to Tregs.

**Table 1:** Patient dog characteristics and clinical outcomes.

Case	Breed	Age	Sex	Tumor		Period of treatment	Survival Time
				Site	Cell type		
**1**	Bulldog	10	F	Shoulder	Nonepitherial malignant tumor	198 days	230 days
**2**	French Bulldog	9	F	Lung metastasis	Renal cell carcinoma	254 days	260 days
**3**	Labrador Retriever	14	M	Humeral bone	Squamous cell carcinoma	84 days	119 days
**4**	Yorkshire Terrier	9	F	Back trunks (multiple)	Malignant fibrous histocytoma	56 days	>800 days

**Fig 14:**
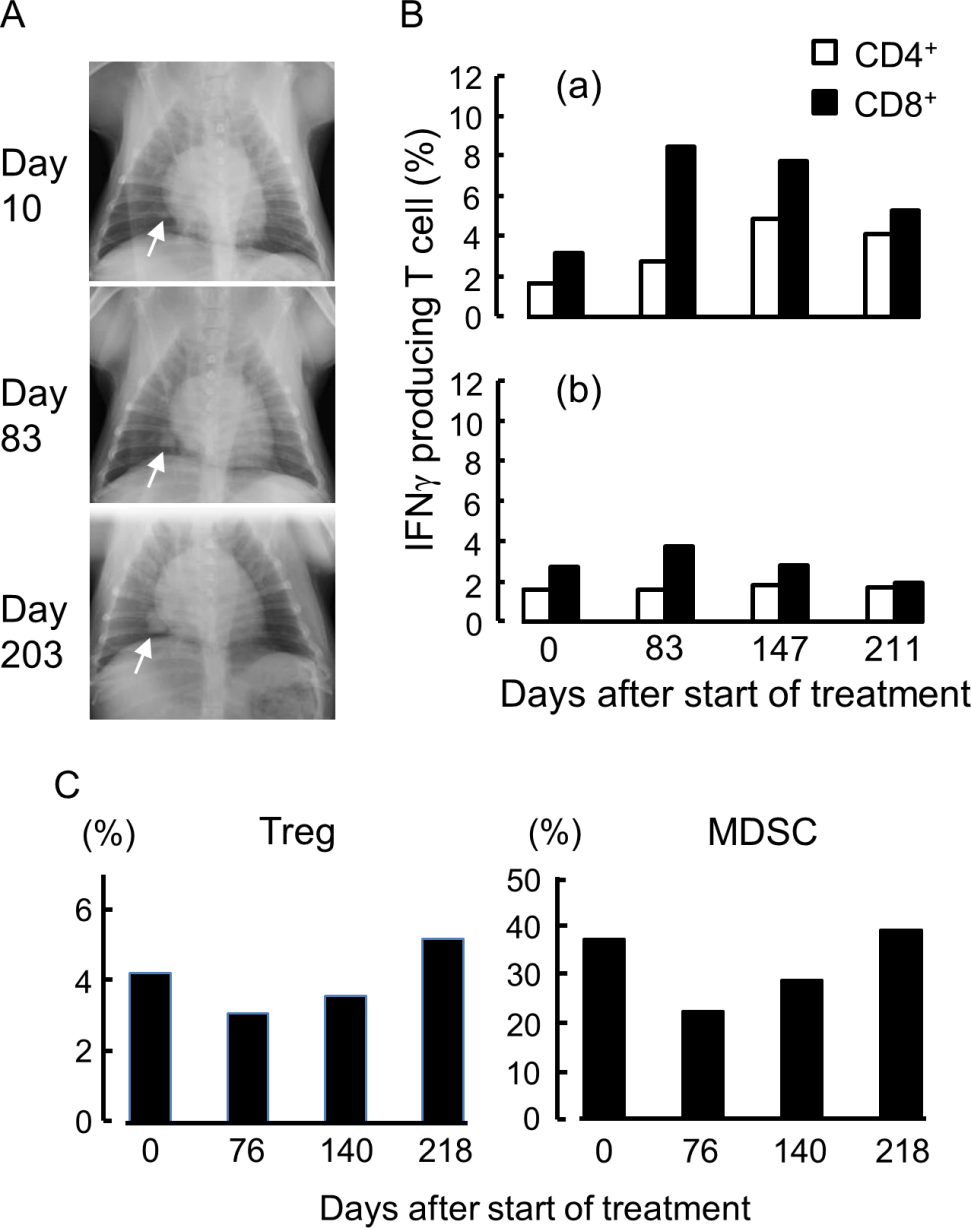
The therapeutic and analytic results of case no. 2. (A) X-ray image of breast at the indicated point of treatment. Arrows indicate metastatic lesions of the same location. (B) Responses of T cells collected from the PB at the points indicated. (a) and (b), same as Fig 13C. (C) Percentage of Treg and MDSC in PB at the points indicated.

Case no. 2 was affected by renal cell carcinoma (S5 Fig). The tumor was removed by operation, but as shown in Fig 14A, metastases were found in the lung. For this patient, the h11c-treated DCs were s.c. injected weekly, and IFNγ was s.c. injected three times per week. Growth of the metastatic tumors was not inhibited (Fig 14A), and the patient died on day 260 due to breathing problems. The TRTCs had increased by day 83, whereas decreased gradually thereafter (Fig 14B). In contrast, Tregs and MDSCs had decreased by the day 76, whereas gradually increased thereafter (Fig 14C).

**Fig 15:**
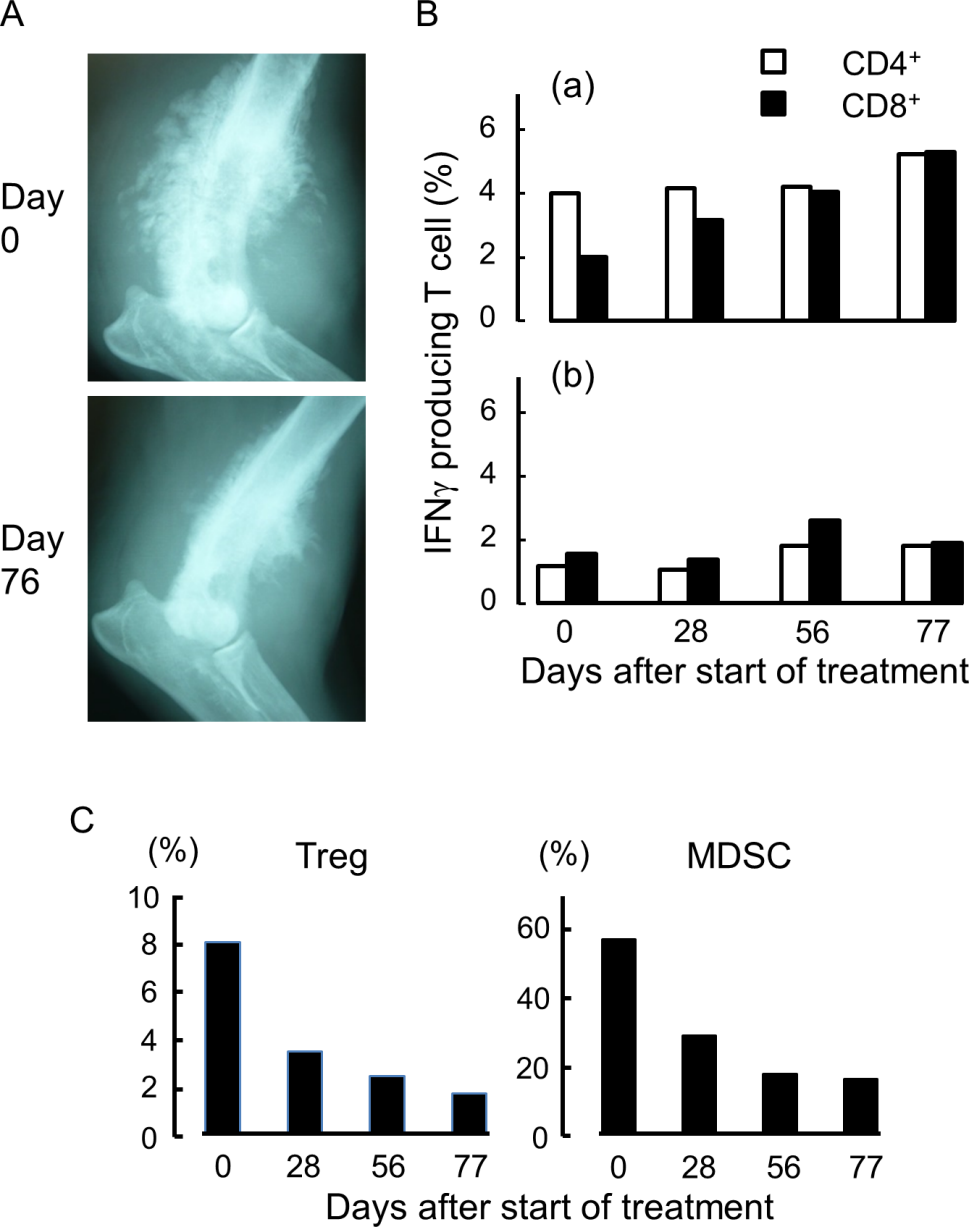
The therapeutic and analytic results of case no. 3. (A) The X-ray image of radial bone before (day 0) and after the 10th treatment (day76). (B) Responses of T cells collected from the PB at the points indicated. (a) and (b), same as Fig 13C. (C) Percent of Treg and MDSC in the PB at the points indicated.

Case no. 3 had a tumor comprising squamous cell carcinoma in the left humeral bone (Fig 15A and S6 Fig). For this patient, h11c-treated DCs and IFNγ were injected into subcutaneous tissue close to the affected site. After 10 cycles of the treatment, the tumor was somewhat smaller (Fig 15A). The TRTCs gradually increased with time (Fig 15B). In contrast, Tregs and MDSCs decreased with time (Fig 15C). Unfortunately, this patient suffered unrelated renal disorder from day 84, and died on day 119.

**Fig 16:**
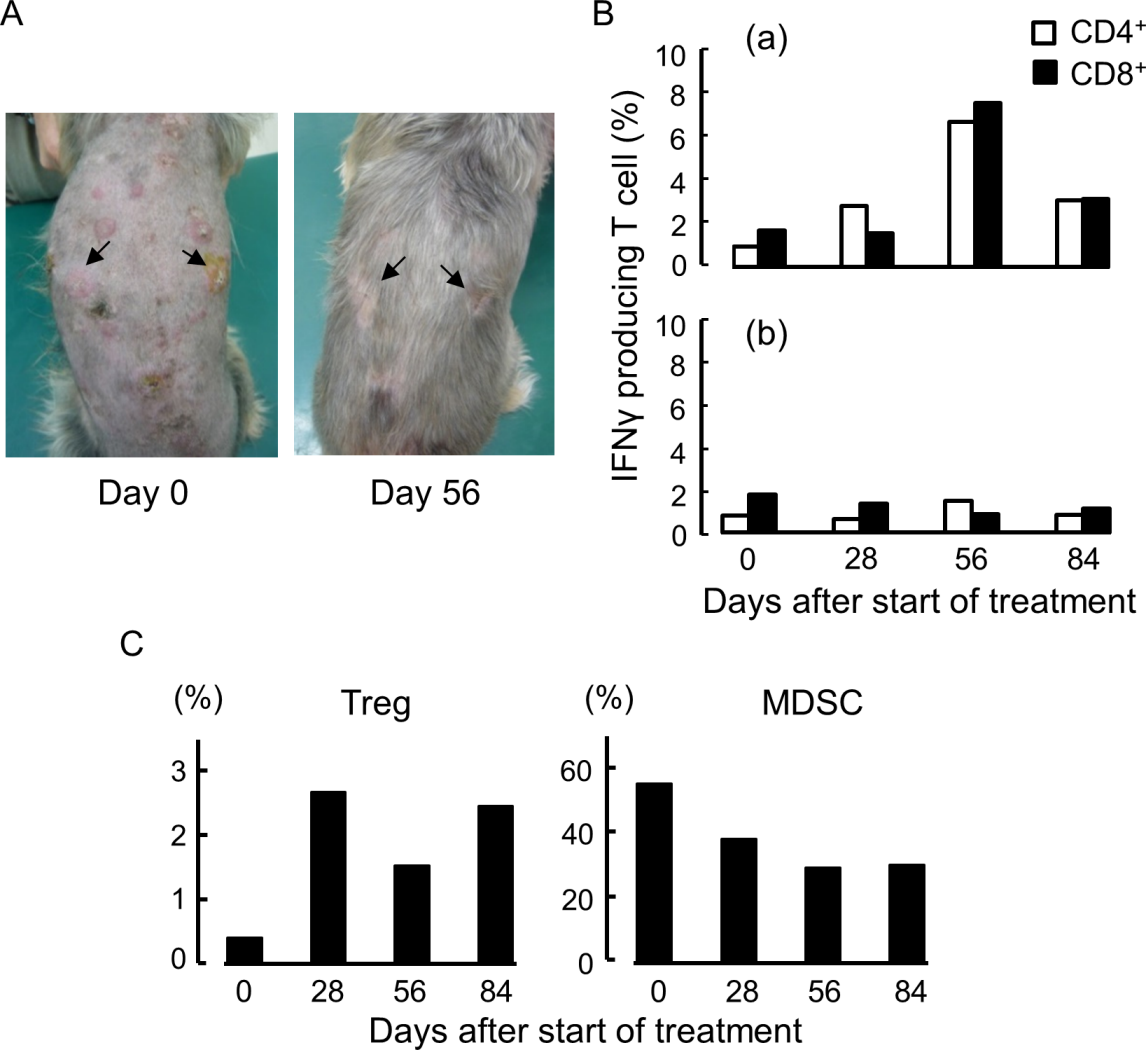
Therapeutic and analytic results for case no. 4. (A) Tumor appearance before and after the 8th treatment (day 56). (B) Responses of T cells collected from the PB at the points indicated. (a) and (b), same as Fig 13C. (C) Percent of Treg and MDSC in the PB at the points indicated.

Case no. 4 had multiple tumors of the nodular type on the back (Fig 16A). Histologically, many histiocytic cells with significant atypical nucleus were observed, and diagnosed as indicating a malignant fibrous histiocytoma (S7 Fig). The h11c-treated DC and IFNγ were injected weekly into two of the tumors indicated by arrows. After eight cycles of this treatment, the treated tumors, and also the other tumors on the back had disappeared completely. No recurrence was subsequently observed, over 800 days. The TRTCs increased upon repeating the treatment, and was greatest at the end of the treatment (Fig 7B). Interestingly, a relatively high response was still observed 28 days after the treatment had stopped. The percentage of Tregs was unrelated to the clinical outcome. But MDSCs nevertheless decreased with time (Fig 7C).

## Discussion

Because of their high efficiency in initiating and activating immune responses, DCs should be a potent adjuvant for tumor immunity. Nevertheless, treatments using DCs expressing the tumor antigens have not generally been successful, especially in cases of metastasis in the internal organs [28]. In humans, additional treatments have therefore recently been performed to enhance DC-based immunotherapy. These include the enhancement of DC function by TLR agonist [29], the amelioration of immune status by inoculation of cytokines to encourage the effector cells [30–32], and the control of suppressor cells such as MDSC [33,34] and Tregs [34,35]. In the present study, we studied all three types of treatment using h11c, IFNγ and COX2-I.

We recently found that IFNγ, together with DCs, induces satisfactory clinical outcomes against surface tumors of the dog in the small size or early stage [12]. However, it revealed thereafter that the DC plus IFNγ treatment still not enough to elicit effective clinical outcomes against malignant tumor in the large size or progressive stage and metastatic tumors in which DCs and IFNγ is difficult to be directly injected. The h11c was designed to bind selectively to human CD11c and activate CD11-expressing DC, and was reported to selectively bind to mouse DC [11]. This property is important to prevent injurious responses caused by activation of macrophages or T cells, and to significantly elicit immune responses against antigens expressed by DCs. As well as the activation of DCs, we newly found that h11c significantly enhanced antigen presentation and antigen-specific CTL-induction by DCs. Moreover, h11c had a significantly higher affinity for canine DCs, and activated them as well as human and murine DCs. In the mouse models of either surface or visceral tumor, the h11c-treated DCs significantly suppressed tumor growth and elicited significantly higher survival. Moreover, the h11c-DCs significantly exerted to reduce monocytic MDSCs. Based on these results, h11c is expected to be a promising tool towards a successful clinical outcome. The COX2-I treatment, which was reported to inhibit the generation of MDSC [14], significantly enhanced the DC plus IFNγ treatment in growth suppression of the mouse surface tumor, and elicit significantly higher survival in the mouse visceral tumor model by adding to the h11c-DC plus IFNγ treatment. The combination of h11c-DC, IFNγ and COX2-I treatment significantly reduced not only monocytic MDSCs but also granulocytic MDSCs. In addition, the combination of treatments significantly enhanced activity of DCs for eliciting immune responses against tumors. Furthermore, the combination induced immune status in tumors to facilitate to remove tumor cells by immune cells. Taking these results together, the combination of h11c-DC, IFNγ and COX2-I treatment was expected to be highly effective in enhancing clinical outcome against both surface and visceral tumors.

In the dog clinical study, using the combination treatment, we challenged to treat surface tumors of larger size (the case no. 1) or systemically diffused (the case no. 4), or metastatic tumors (the cases no. 2 and 3). In three of the four cases the combined treatment significantly suppressed the growth of tumors. In these cases TRTCs in PB increased and inversely almost correlate with the tumor volume. It is noteworthy that not only MDSC but also Tregs decreased in correlation with the tumor volume, although the underlying mechanism was not clear. In case no. 1 the tumor volume had increased by day 49, regardless of the treatment, but decreased rapidly thereafter. The TRTCs increased during this period, but Tregs and MDSCs in the PB decreased. From these results it appears that the early growth of the tumor is due to partially inflammatory swelling caused by immune responses against the tumor. On the other hand, the combined the combined treatment permitted the growth of lung metastatic tumors (the case no, 2). However, the TRTCs level markedly increased in the beginning of the treatment. Although gradually decreased with time, it kept higher than that before treatment by the end period. The level of the Tregs and MDSCs decreased at the beginning, but increased gradually thereafter, and reached higher than that before treatment. Based on these results, it is therefore suggested that potent control of these suppressor cells is a key to succeed in treatment against visceral metastatic tumors. Hence, to control these suppressor cells in the dog, we are planning to use additional reagents such as sunitinib [34]. Overall, these results indicate that the combination of h11c-DC, IFNγ and COX2-I effectively induces strong responses to overcome tumor growth.

## Supporting information

S1 Fig The appearance of tumor formation by LM8 and CT26.WT cells. (TIF)

S2 Fig Representative FCM profile concerning costimulatory molecule expression of DCs. (TIF)

S3 Fig Representative FCM profile concerning cytokine expression of DCs. (TIF)

S4 Fig The histological profile of the tumor in the case no. 1. (TIF)

S5 Fig The histological profile of the tumor in the case no. 2. (TIF)

S6 Fig The histological profile of the tumor in the case no. 3. (TIF)

S7 Fig The histological profile of the tumor in the case no. 4. (TIF)
