# Supplementary figures and images for: Development of effective tumor immunotherapy using a novel dendritic cell–targeting Toll-like receptor ligand
Source: PLoS One. 2017 Nov 30;12(11):e0188738. doi: 10.1371/journal.pone.0188738 (PMC5708771; doi:10.1371/journal.pone.0188738)

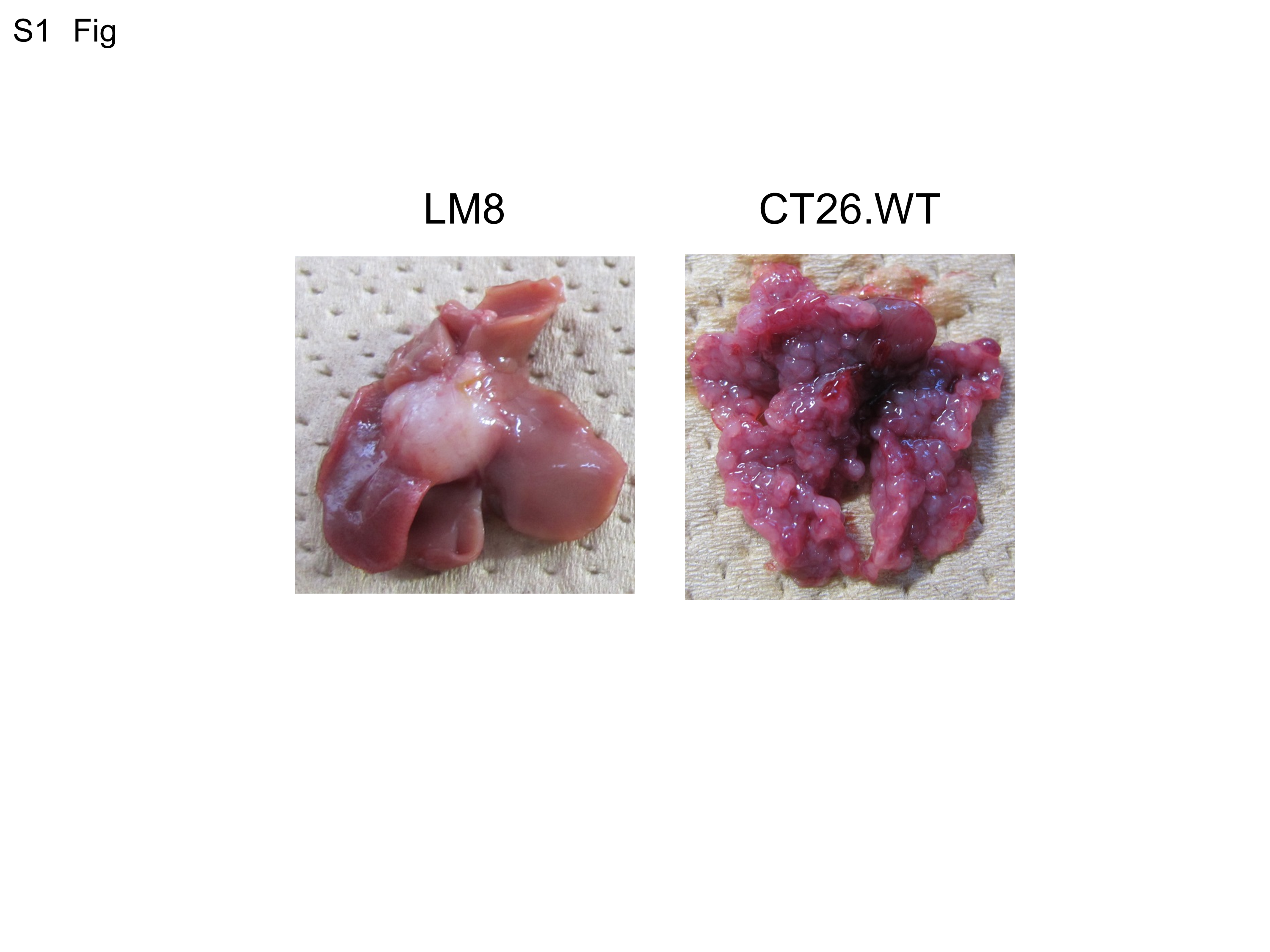

Supplement: S1 Fig — (TIF) [file pone.0188738.s001.tif]

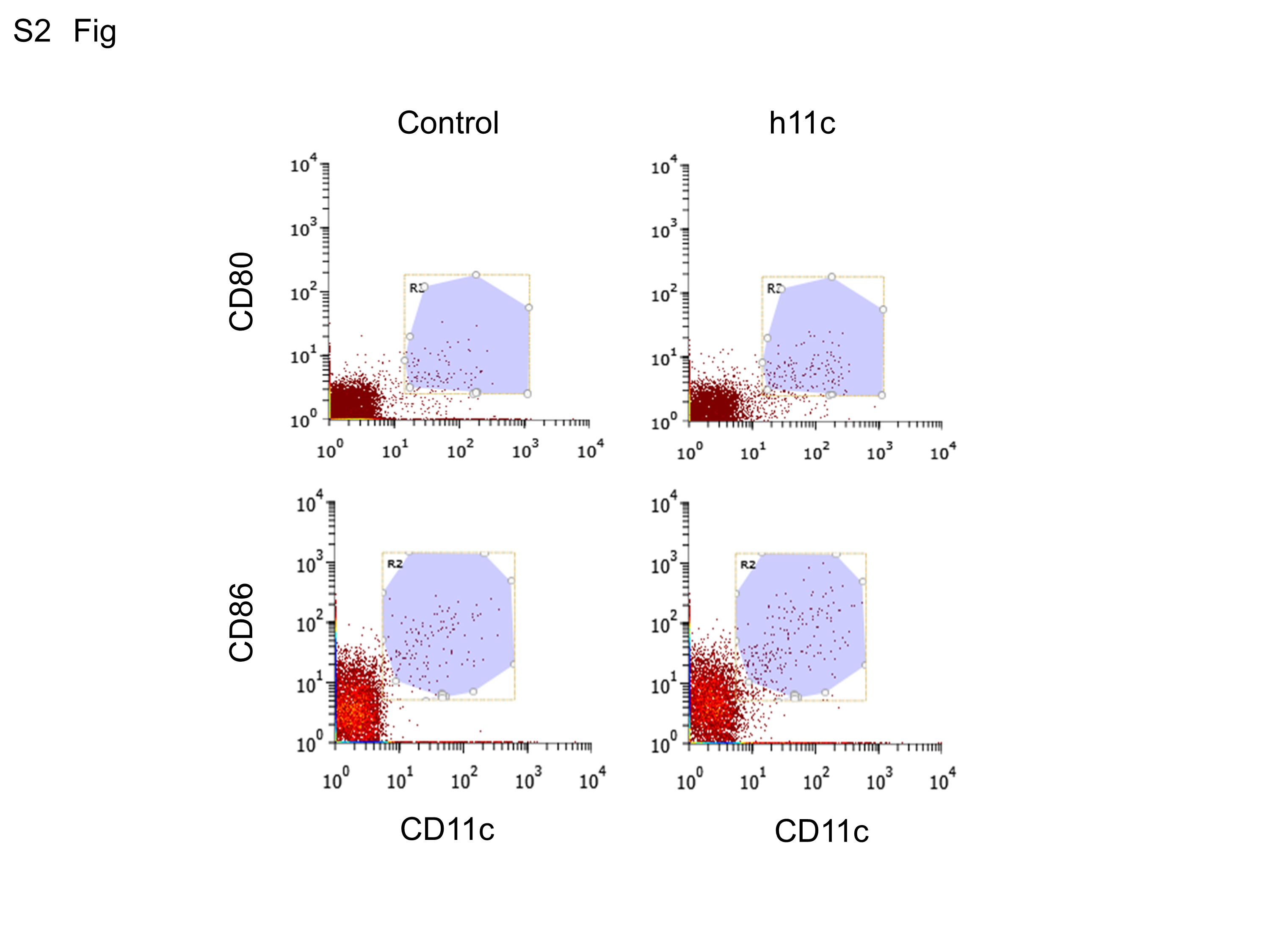

Supplement: S2 Fig — (TIF) [file pone.0188738.s002.tif]

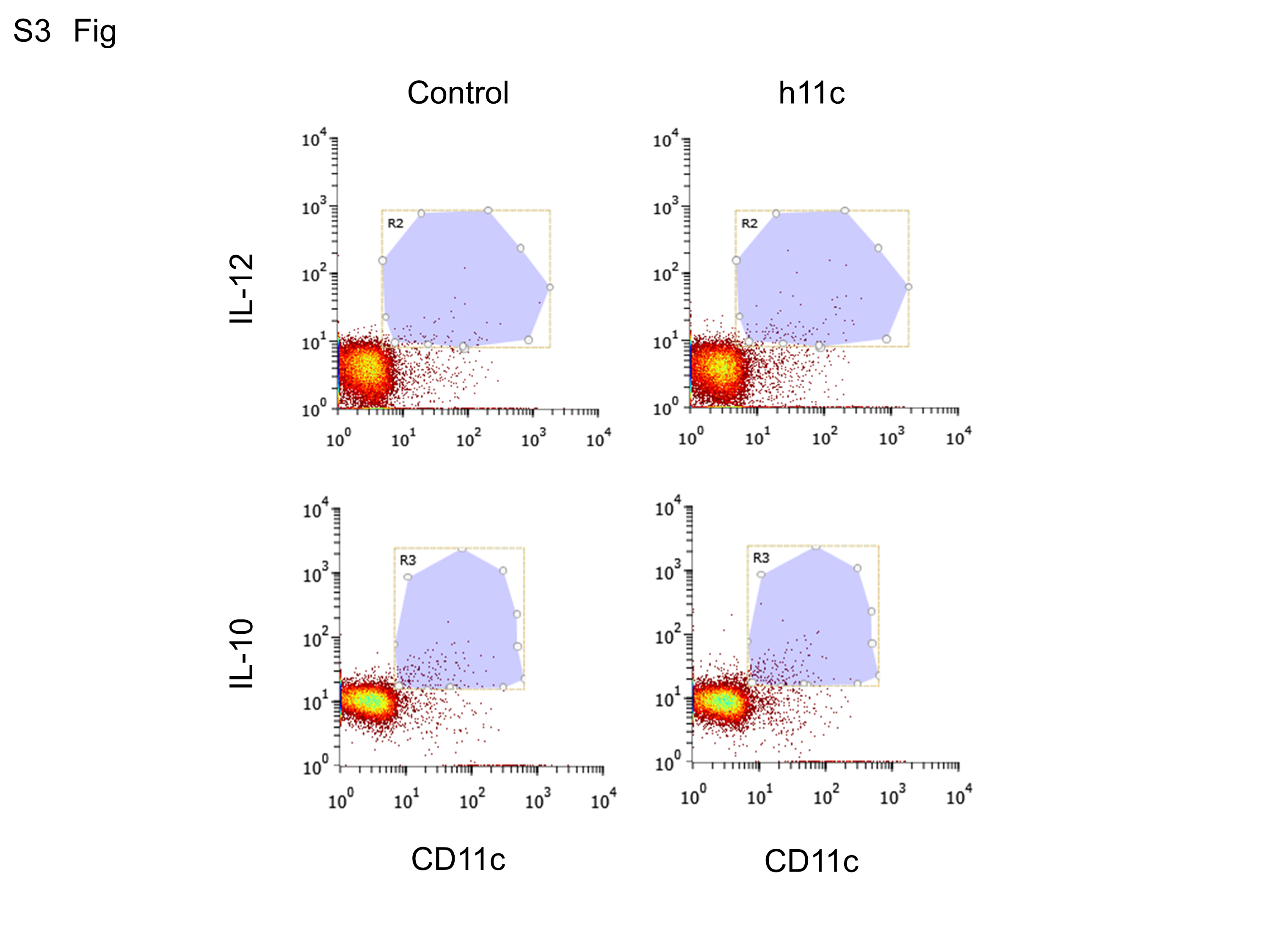

Supplement: S3 Fig — (TIF) [file pone.0188738.s003.tif]

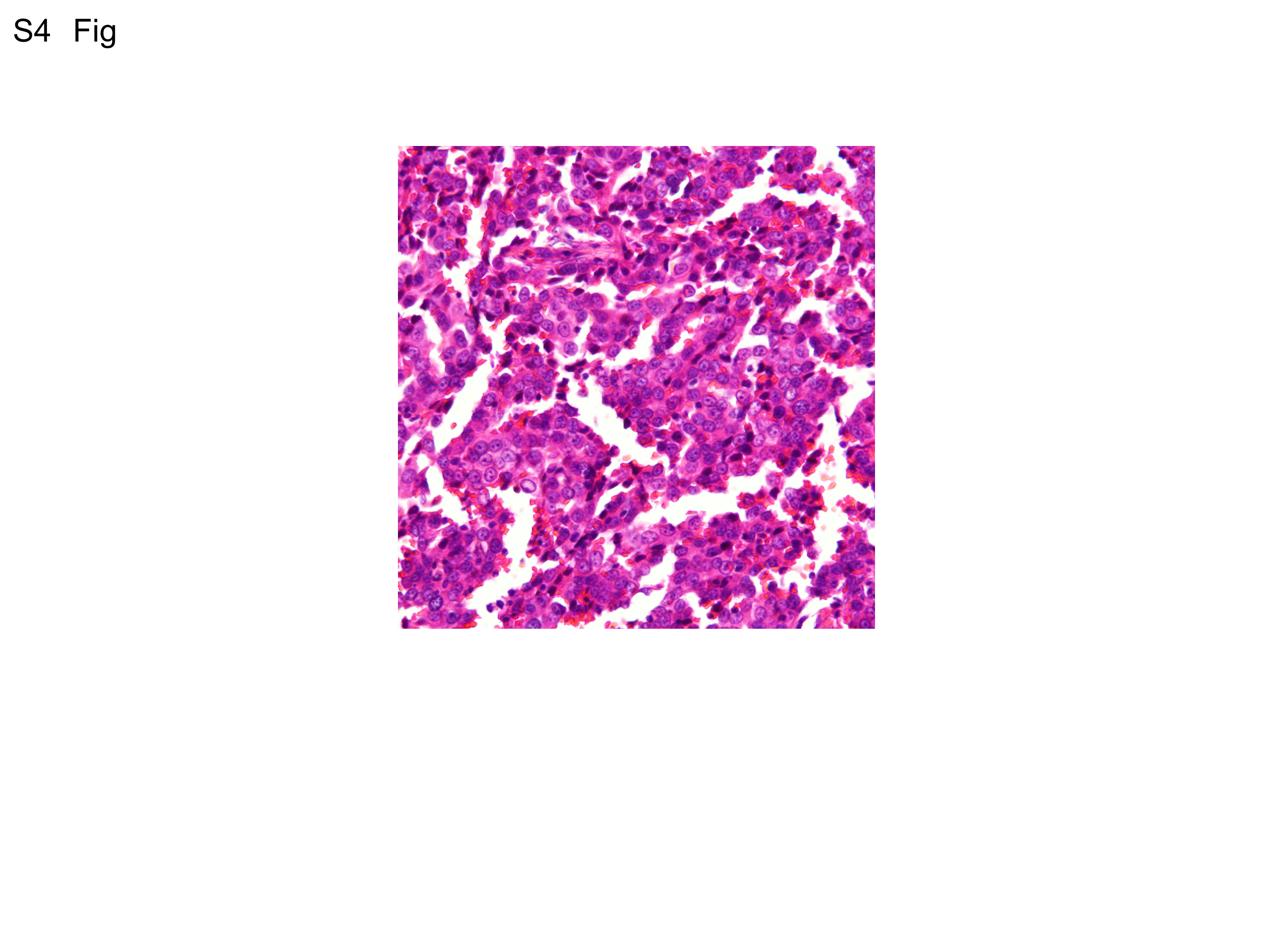

Supplement: S4 Fig — (TIF) [file pone.0188738.s004.tif]

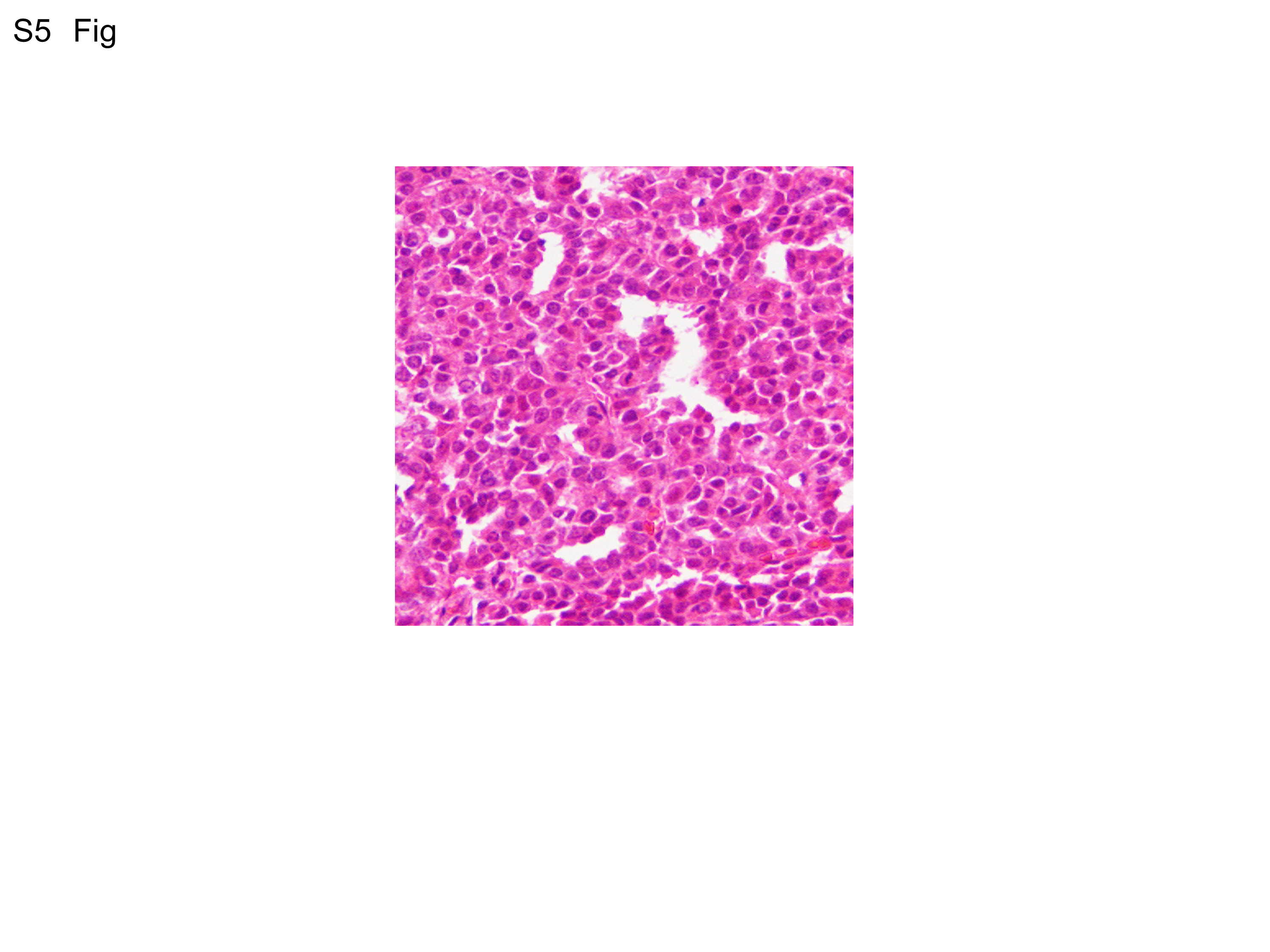

Supplement: S5 Fig — (TIF) [file pone.0188738.s005.tif]

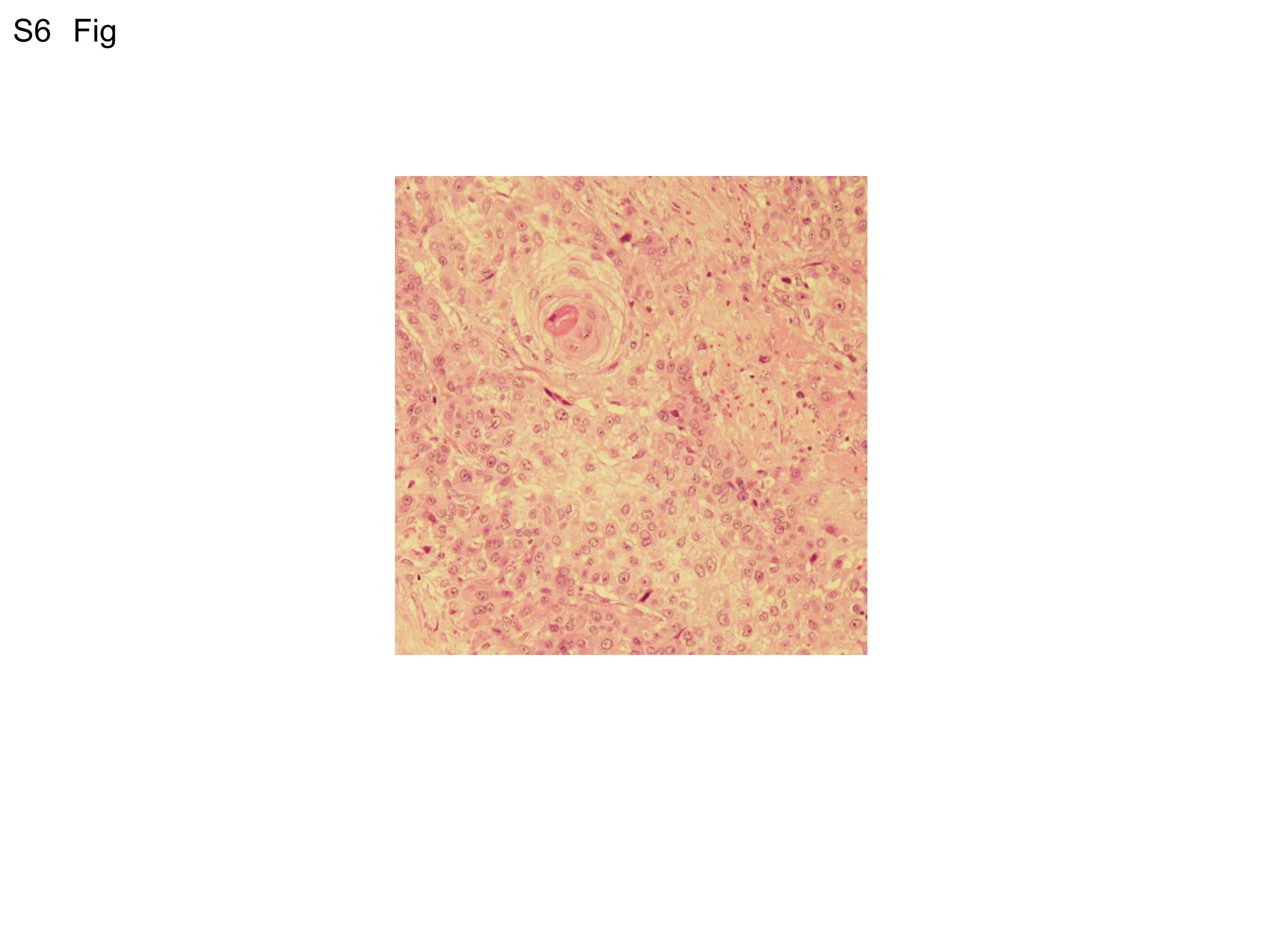

Supplement: S6 Fig — (TIF) [file pone.0188738.s006.tif]

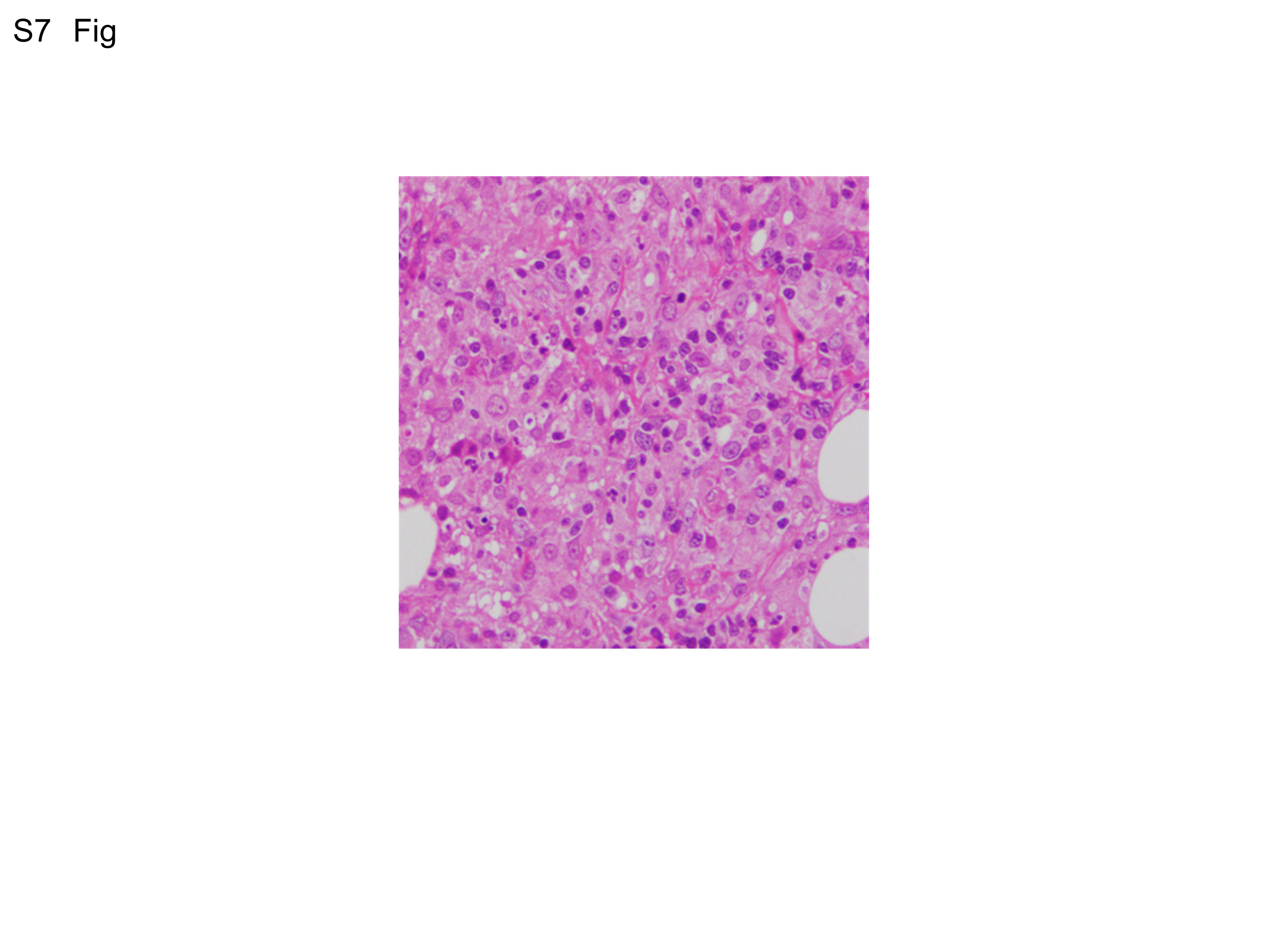

Supplement: S7 Fig — (TIF) [file pone.0188738.s007.tif]
